# *Candida albicans* Commensalism and Pathogenicity Are Intertwined Traits Directed by a Tightly Knit Transcriptional Regulatory Circuit

**DOI:** 10.1371/journal.pbio.1001510

**Published:** 2013-03-19

**Authors:** J. Christian Pérez, Carol A. Kumamoto, Alexander D. Johnson

**Affiliations:** 1 Department of Microbiology and Immunology, University of California, San Francisco, San Francisco, California, United States of America; 2 Department of Molecular Biology and Microbiology, Tufts University, Boston, Massachusetts, United States of America; Duke University Medical Center, United States of America

## Abstract

Systemic, life-threatening infections in humans are often caused by bacterial or fungal species that normally inhabit a different locale in our body, particularly mucosal surfaces. A hallmark of these opportunistic pathogens, therefore, is their ability to thrive in disparate niches within the host. In this work, we investigate the transcriptional circuitry and gene repertoire that enable the human opportunistic fungal pathogen *Candida albicans* to proliferate in two different niches. By screening a library of transcription regulator deletion strains in mouse models of intestinal colonization and systemic infection, we identified eight transcription regulators that play roles in at least one of these models. Using genome-wide chromatin immunoprecipitation, we uncovered a network comprising ∼800 target genes and a tightly knit transcriptional regulatory circuit at its core. The network is enriched with genes upregulated in *C. albicans* cells growing in the host. Our findings indicate that many aspects of commensalism and pathogenicity are intertwined and that the ability of this microorganism to colonize multiple niches relies on a large, integrated circuit.

## Abstract 2

The identification of regulators, circuits, and target genes employed by the fungus *Candida albicans* to thrive in disparate niches in a mammalian host reveals interconnection between commensal and pathogenic lifestyles.

## Author Summary

Our skin and mouth, as well as our genital and gastrointestinal tracts, are laden with microorganisms belonging to all three domains of life (bacteria, archaea, and eukaryotes). Much of the time these commensal microorganisms are not only harmless but provide advantages to us. However, when the host's defenses are compromised, some members of the normal flora, such as the fungus *C. albicans*, can cross the host's protective barriers and colonize virtually every internal organ causing life-threatening conditions. The environment found in the bloodstream and internal organs is presumably distinct from the mucosal surfaces where our flora typically resides. Whether opportunistic pathogens such as *C. albicans* rely on common or separate gene repertoires to thrive in each of these locales is largely unknown. To address this question we carried out genetic screens in mouse models that recapitulate niches where *C. albicans* thrives and used genome-wide experimental approaches to uncover the genes required to proliferate in each environment. In fact, the ability of *C. albicans* to colonize disparate niches within a mammalian host relies on a large, integrated circuit. Our observations suggest that at least some key gene circuits are not dedicated to one niche or another. Rather, thriving in various locales of the host seems to involve the complex regulation of multiple processes, which may allow *C. albicans* to adjust to different environments.

## Introduction

Mammalian mucosal surfaces harbor trillions of microorganisms from all three domains of life [1]–[4]. While most of these microorganisms are harmless (or beneficial) to their host, a few of them are able to cross the host's protective barriers and colonize internal organs that offer little apparent resemblance to the microbe-laden mucosal surfaces. Indeed, many of the life-threatening infections in humans are caused by the very same bacterial or fungal species that typically compose our own microbiota. A hallmark of these opportunistic pathogens therefore is their ability to proliferate in disparate niches within the host. It remains an open question, however, whether the repertoire of genes that enables such pathogens to thrive in the host varies from one niche to the other.

In this paper we investigate the case of *C. albicans*, the most prominent fungal species living on mucosal surfaces—particularly in the gastrointestinal (GI) tract—of warm-blooded animals [5]–[7]. While it is a member of the normal human microbiota, *C. albicans* can also cause mucosal disease in healthy hosts or produce systemic infections and colonize internal organs in people who have received surgical implants or whose immune systems have been compromised, such as AIDS patients or individuals receiving chemotherapy. Deep-seated infections often result in life-threatening conditions. In addition to the status of the host immune system [8],[9], the outcome of the *C. albicans*–host interaction depends on various products and functions encoded in the genome of the fungus, as multiple gene deletions render *C. albicans* avirulent in both mucosal and invasive animal models (reviewed in [10]). For example, the production of extracellular hydrolases [11], the ability to switch between yeast and filament forms [12]–[14], and the production of small molecules [15] are all necessary for *C. albicans* to proliferate as either commensal or pathogen. The recent generation of relatively large collections of gene deletion mutants makes it now possible to carry out systematic and unbiased searches for genes and cellular functions employed by *C. albicans* to thrive in the host.

To begin to dissect the repertoire of genes that enable *C. albicans* to colonize the mammalian GI tract and determine whether these genes also play a role during systemic infection, we screened a collection of 77 *C. albicans* transcription regulator (TR) mutants in mouse models that recapitulate these two niches. We focused on TRs because transcriptional circuits are central to the regulation of many biological processes. The subset of TRs we screened was chosen because their deletion in *C. albicans* produces neither significant growth defects nor anomalous colony morphologies under any of 55 different laboratory growth conditions that have been tested [16]. Thus, we expected to maximize the identification of regulators “dedicated” to biological processes directly connected to *C. albicans* proliferation in the host. This approach also minimized the retrieval of mutants with either large pleiotropic effects or with fitness defects not specific to life in the host.

Here we report the identification of eight *C. albicans* TRs required for GI tract colonization, systemic infection or both. We elucidate the transcriptional circuitry controlled by these regulators using genome-wide experimental approaches, and find that the resulting network is enriched with genes that are upregulated when *C. albicans* grows in the host. Five of the identified TRs form a highly interconnected core network that regulates determinants of GI tract colonization as well as systemic infection indicating that both types of growth in the host require common circuitries. We find that cell surface remodeling and the acquisition of carbon and nitrogen are salient among the functions that *C. albicans* requires to proliferate in the host. Finally, we demonstrate that several of the gene products regulated by the identified TRs are in fact required for intestinal colonization or for systemic infection. Thus, the use of TRs as genetic entry points, combined with full-genome molecular biology methods, can identify regulators, circuits, and target genes needed explicitly for *C. albicans* to colonize different niches of mammalian hosts.

## Results

### Genetic Screen to Identify *C. albicans* Transcription Regulators Required to Colonize the Murine Gastrointestinal Tract

**Figure 1:**
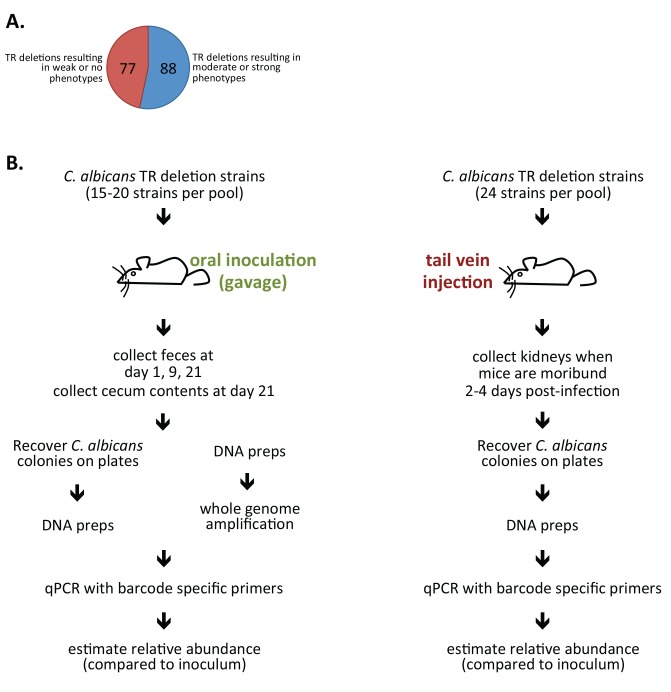
Gastrointestinal tract colonization and systemic infection screens. (A) About 45% of *C. albicans* TR deletion strains (*n* = 165) display no significant growth or colony morphology phenotype under any of 55 different laboratory growth conditions [16]. We used this subset of TRs (*n* = 77) to carry out genetic screens in mouse models that recapitulate niches where *C. albicans* thrives. (B) Schematic of the GI tract colonization and bloodstream infection screens. In the GI tract colonization model we used two different approaches to recover DNA from the fecal pellets and intestinal contents: (1) DNA was prepared directly from the samples, or (2) the samples were first plated and the DNA was purified from yeasts scraped off the plates. Similar results were obtained with samples that were processed directly or that were plated.

Sequence-specific DNA binding proteins that regulate transcription, or TRs, are major elements within the gene network of an organism. They are pivotal in orchestrating responses to external cues and in maintaining internal homeostasis in the face of fluctuations in the environment. TRs are thus likely to be critical components of the gene network that underlies the ability of *C. albicans* to inhabit the host. Our laboratory recently constructed a collection of 165 *C. albicans* TR homozygous deletion mutant strains consisting of two independently generated, fully vetted isolates of each deletion [16]. About 45% of the *C. albicans* TR deletion strains display no significant growth or colony morphology phenotype under any of 55 different laboratory growth conditions (Figure 1A) [16], raising the possibility that their function may be revealed only in the context of the host. Hence, we focused on this subset of TRs (*n* = 77) to carry out genetic screens in mouse models that recapitulate niches where *C. albicans* thrives (Figure 1B).

**Figure 2:**
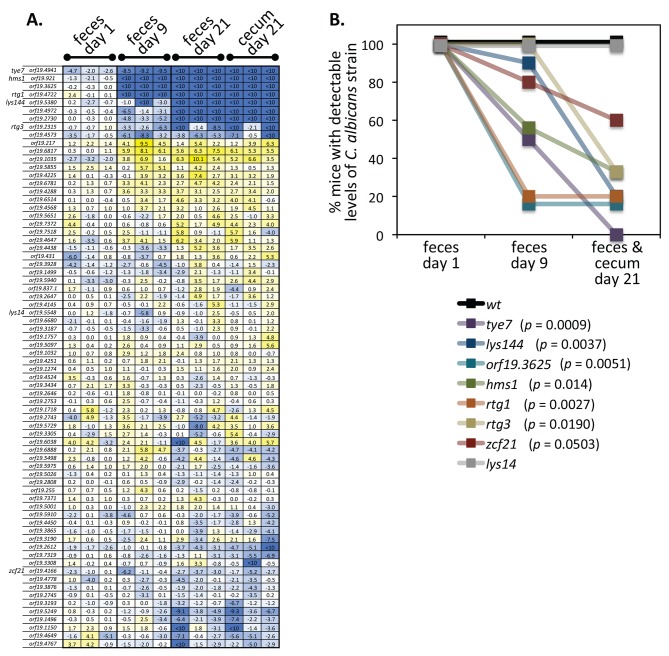
Identification of regulators that govern *C. albicans* proliferation in the murine gastrointestinal tract. (A) Log_2_ (recovered/input) values for *C. albicans* TR mutants at different time points after oral inoculation by gavage in three mice. The order in which the mutants are displayed reflects hierarchical clustering. Color intensity indicates reduction (blue) or accumulation (yellow). An independent isolate of each of the top eight mutants in the panel was tested in an iteration of the screen. *orf19.4972* and *orf19.2730* did not reproduce the effect. (B) The percentage of mice with detectable levels of various *C. albicans* mutants at different time points after gavage are plotted and their *p*-values (logrank test) are indicated.

To minimize the number of animals required to screen the mutant strain library, we adopted the signature-tagged mutagenesis technique that our laboratory has successfully used to identify virulence factors in *C. albicans* [15] and has been employed in other fungi [17] and bacteria [18] as well. We used a mouse model of intestinal colonization in which immunocompetent antibiotic-treated Swiss Webster mice are orally inoculated with *C. albicans* by gavage [19],[20]. While mice do not appear to be natural hosts for *C. albicans* but for the closely related yeast *C. tropicalis* [1], the murine GI colonization model has been adopted as the standard in the field to evaluate *C. albicans* commensalism [21]–[23]. We assayed pools of 15–20 signature-tagged mutant strains; the relative abundance of the strains recovered from feces (at 1, 9, and 21 d post-inoculation) or intestinal contents (at day 21 when mice were euthanized) compared to the inoculum was determined by real time PCR (using primers to the signature tags) as described [15]. We were able to confidently monitor 72 *C. albicans* strains over the course of the experiment in three mice each. The level of depletion or accumulation of each mutant relative to the inoculum is shown in Figure 2A. We found that ∼1,000-fold reduction with respect to the inoculum is the limit of accurate detection for most strains in this assay (that is, a log_2_ value of about −10). While the actual values can vary from mouse to mouse, they do show a high degree of consistency across samples (e.g., intestinal contents versus fecal pellets at day 21) and across time points (e.g., mutants that became undetectable at day 9 remained so at day 21). The weight and body condition of all inoculated mice were closely monitored throughout the experiment; no differences were observed between inoculated and control animals.

**Figure 3:**
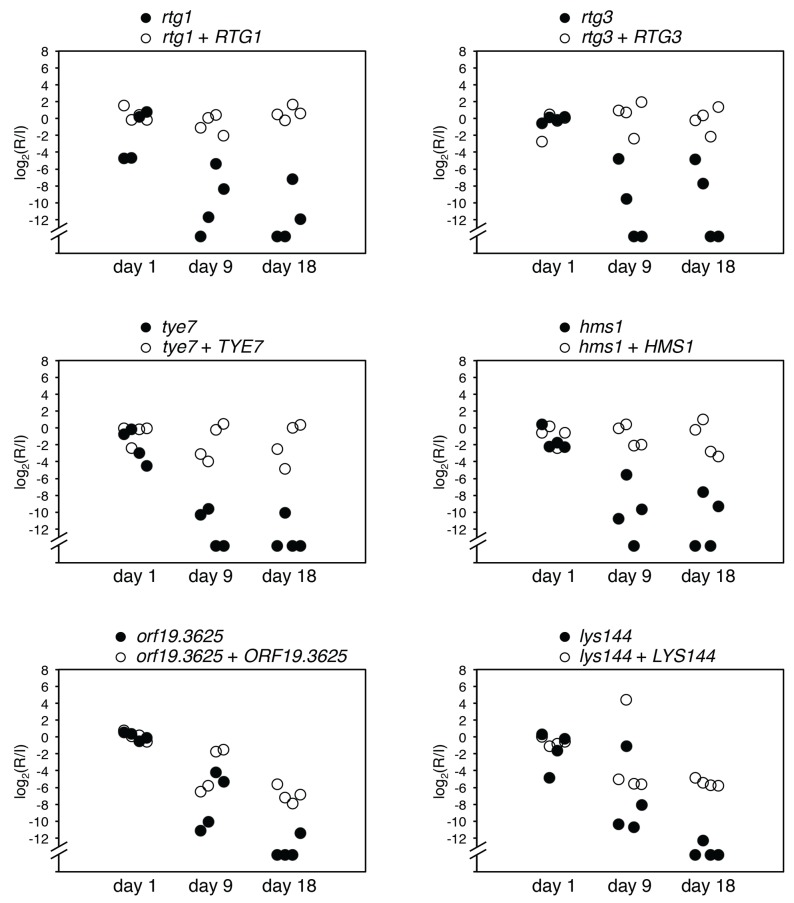
Wild-type genes restore ability to endure in the intestine to mutants impaired in colonization. Two-strain oral infections were carried out comparing each mutant to a gene add-back strain in four mice. qPCR was used to estimate the abundance of each strain. Shown are the log_2_ (recovered/inoculum) values per mouse for the mutant (closed circles) and the add-back (open circles) strains.

For each mutant that showed a severe defect (i.e., those that fell below the level of detection of the assay at day 9 or 21 post-inoculation) we tested an independently constructed deletion of the same gene. We also re-tested in this assay all the mutants that showed defects in our second screen, a systemic infection model (see below). We focused on mutants with large effects (>1,000-fold reduction relative to the inoculum) and, to facilitate the statistical analysis, we converted the data to binary mode: presence or absence. The data displayed in this manner are shown in Figure 2B. Each mutant shown here was evaluated in at least six mice, with both independent isolates producing consistent results. Based on these criteria, six TR deletion mutants (*tye7*Δ/Δ [*orf19.4941*], *rtg1*Δ/Δ [*orf19.4722*], *rtg3*Δ/Δ [*orf19.2315*], *lys144*Δ/Δ [*orf19.5380*], *hms1*Δ/Δ [*orf19.921*], and *orf19.3625*Δ/Δ) showed significant, large impairments in GI tract colonization (*p*<0.02) while *zcf21*Δ/Δ (*orf19.4166*) has a weaker defect (*p* = 0.0503). To verify that the phenotype is due to the deleted gene, we reintroduced an ectopic copy of the wild-type allele back into each mutant and found that it was able to restore colonization at least partially in all mutants (Figure 3). As noted previously, the mutants have no growth defect in standard laboratory conditions and none of the six regulators had been previously implicated in intestinal colonization.

Of the six regulators, *TYE7* is known to control carbohydrate metabolism in *C. albicans* [24] and contributes to the cohesiveness and correct hyphal formation of biofilms [25], while *HMS1* has recently been reported to be required for *C. albicans* morphogenesis at elevated temperatures (42°C) [26]. Beyond the initial phenotypic screening describing the TR deletion collection [16], no function has been ascribed to any of the other TRs in *C. albicans*, although in *Saccharomyces cerevisiae RTG1* and *RTG3* are key regulators of the mitochondrial retrograde response (described below) [27],[28].

### Partial Overlap between the Set of TRs Required to Colonize the GI Tract and TRs Playing Roles in Systemic Infection

**Figure 4:**
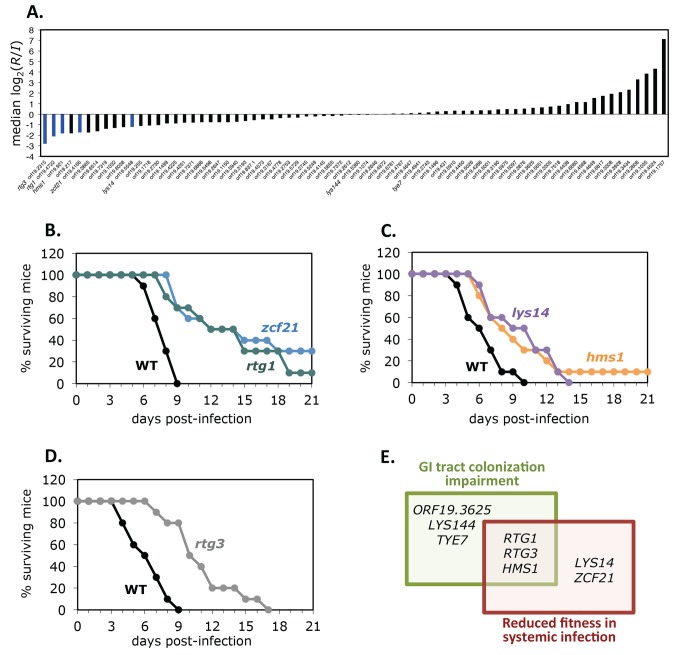
Identification of regulators required for *C. albicans* systemic infection. (A) Results of the systemic infection screen. Bars represent median log_2_ (recovered/input) values. (B–D) Virulence analysis of selected mutant deletion strains in monotypic infections. Ten BALB/c mice were infected with wild-type *C. albicans* or one of the mutant strains by tail vein injection. We used 5.2×10^5^ cells of each strain per infection. Mice were monitored daily and sacrificed when moribund. The logrank test was used for statistical analysis: *p*<0.0001 for *zcf21*; *p* = 0.0008 for *rtg1*; *p* = 0.0203 for *hms1*; *p* = 0.0166 for *lys14*; and *p* = 0.0001 for *rtg3*. (E) Summary of the TRs displaying phenotypes in the mouse models that we evaluated.

To determine whether a given *C. albicans* TR play a role in colonization of the GI tract as well as during systemic infection, we evaluated the fitness of the same set of 77 TR deletion mutant strains in a mouse model of disseminated candidiasis. We chose tail vein injection because this model has been adopted as the standard in the field to assess *C. albicans* virulence. Pools of 24 signature tagged mutant strains were assayed, and the relative abundance of the strains recovered from the kidneys of moribund BALB/c mice (2–4 d post-infection) (Figure 1B) was compared to that in the infecting inoculum using real time PCR [15]. For about two-thirds of the mutant strains, two independent isolates were evaluated. Only one isolate was tested for the other third. Every strain was assayed in at least four mice. The results obtained for all the mutants are shown in Figure 4A and Table S1. To consider a mutant strain as having fitness defect, both isolates had to exhibit consistent results (none of the mutants for which only a single isolate was tested showed any defect). Based on this criterion, the screen revealed five TR deletion mutants (*rtg1*Δ/Δ, *rtg3*Δ/Δ, *zcf21*Δ/Δ, *lys14*Δ/Δ [*orf19.5548*], and *hms1*Δ/Δ) with reduced fitness (*p*<0.05). These five mutants were retested individually and showed reduced virulence in single (as opposed to pooled) tail vein infections when time to illness was monitored (Figure 4B–4D). Careful scrutiny of the other three TR deletion mutants with defects in GI tract colonization (*tye7*Δ/Δ, *lys144*Δ/Δ, and *orf19.3625*Δ/Δ) confirmed that they did not show abnormalities in our systemic infection model (in agreement with this observation, [24] also found no defect for a *tye7* mutant in related models of disseminated candidiasis). In sum, our two in vivo genetic screens uncovered eight TRs playing roles in the proliferation of *C. albicans* within the host. Three of these regulators (*RTG1*, *RTG3*, and *HMS1*) exhibited significant impairment in both GI tract colonization and systemic infection (Figure 4E). *ZCF21* displays a significant fitness anomaly in systemic infection but only a weak and variable defect in GI tract colonization. *LYS14* showed a defect in systemic infection but not GI tract colonization. *ORF19.3625*, *LYS144*, and *TYE7* showed the opposite behavior being required for intestinal colonization but not systemic infection. We excluded *ORF19.3625* from further study because it encodes a putative subunit of a histone remodeling complex and as such it is unlikely to be a specific regulator for a particular set of genes.

### Connecting the Regulators of Intestinal Colonization and Systemic Infection to Target Genes

**Figure 5:**
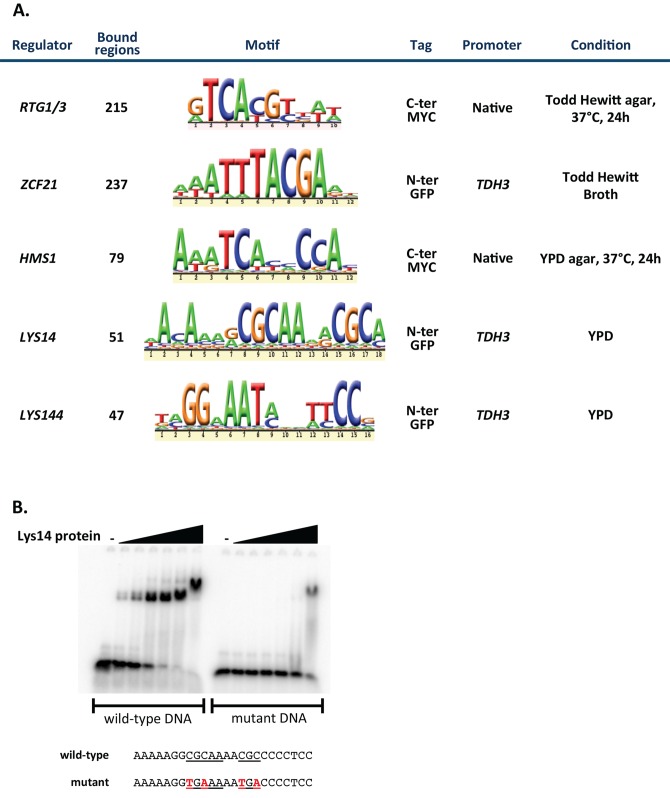
Genome-wide identification of DNA regions bound by TRs. (A) Summary of experimental conditions and results of ChIP. ChIP-chip was carried out for each of the identified regulators under the conditions described. The number of intergenic regions bound by each regulator is indicated. The DNA motifs shown were derived de novo from our ChIP-chip data and are significantly enriched in the bound regions. (B) The *C. albicans* Lys14 protein binds in vitro to the DNA sequence identified by ChIP-chip. The DNA-binding domain of the *C. albicans* Lys14 protein (amino acids 1–236) was N-terminally fused to 6His and to the maltose binding protein, expressed in *Escherichia coli* and purified with Ni-NTA columns. ^32^P-labeled 24-nt DNA fragments (∼0.4 nM) containing the predicted wild-type or mutant Lys14 binding site were incubated with increasing concentrations of purified Lys14 protein (0, 0.039, 0.156, 0.625, 2.5, 10, and 40 nM) for 30 min at room temperature in standard EMSA buffer and resolved in 6% polyacrylamide gels run with 0.5× TGE. The DNA fragment tested corresponds to the *OCH1* promoter. The point mutations that we inserted to disrupt the putative binding site are indicated in red.

To gain insights into the biological processes directed by the seven identified TRs, we determined the genes that they regulate. *TYE7* is the only one of the regulators for which genome-wide data regarding its target genes in *C. albicans* are available (see [24]). Thus, we carried out whole genome chromatin immunoprecipitation followed by array hybridization (ChIP-chip) for the remaining six TRs. As it might be predicted, the conditions typically used to grow *C. albicans* in the laboratory (liquid culture in YPD medium at 30°C) were not optimal to detect either binding of the TRs to their target promoters or changes in the expression of target genes (i.e., in expression arrays comparing wild-type versus TR deletion mutant strains). This was not unexpected because the mutants chosen for the screen have no significant phenotypes when tested under laboratory conditions; therefore, the identified regulators are likely to be active only under specific conditions within the host. To overcome this limitation we constructed fluorescent reporter strains (*yfp* or *gfp* fused to each regulator's native promoter and YFP- or GFP-fused TR proteins) and sought conditions that promoted either the expression or the nuclear localization (in the case of fusion proteins) of the fluorescent reporters. Among the conditions tested were ∼20 different cell culture media, 37°C (the temperature in the host) and the growth of cells on a semi-solid surface (which may mimic growth on the surfaces within the host). Figure 5A summarizes the optimal growth conditions that were chosen to immunoprecipitate each regulator in vitro. In the case of *ZCF21*, *LYS14*, and *LYS144*, we nonetheless had to increase their expression artificially using the *TDH3* promoter to be able to immunoprecipitate them.

Using stringent cutoffs to define statistically significant binding events (see Materials and Methods), we established that the following number of intergenic regions are bound by each regulator: 79 for Hms1, 51 for Lys14, 47 for Lys144, 237 for Zcf21, and 215 for Rtg1 and Rtg3 (Dataset S1). The ChIP-chip profiles of Rtg1 and Rtg3 were identical to each other implying that these two proteins bind to DNA together. Indeed, the *S. cerevisiae* Rtg1 and Rtg3 orthologous proteins are known to form a heterodimer to bind to DNA (reviewed in [29]). Using only the ChIP-chip data, we were able to derive DNA motifs (i.e., *cis*-regulatory sequences) for each regulator (Figure 5A). These sequences were significantly enriched in the bound regions compared to the remainder of intergenic regions (Figure S1). The motif that we derived for Rtg1/3 is similar to the reported binding sequence of their orthologs in *S. cerevisiae* (GTCAC) [29]. Likewise, the motif that we find for Lys144 resembles the reported binding sequence for its closest homolog in *S. cerevisiae*, Lys14 (TCCRNYGGA) [30]. The motif that we derived for Hms1, a member of the basic helix-loop-helix family of TRs, matches the non-E-box consensus binding sequence (ATCACCCCAC) for SREBP1, the prototypical member of the family [31]. Although the Lys14 motif that we generated differs from the sequence recognized by its homolog Lys14 in *S. cerevisiae*, we confirmed by gel mobility shift assays that the purified *C. albicans* Lys14 protein binds in vitro to the sequence that we identified (Figure 5B). (Phylogenetic reconstructions indicate that the closest homolog of *S. cerevisiae LYS14* in *C. albicans* is *LYS144* and not *LYS14*, albeit the current nomenclature implies otherwise. In addition, as described below, *C. albicans LYS144* and *LYS14* have nothing to do with lysine biosynthesis regulation.) We were unable to identify an ortholog in *S. cerevisiae* for *C. albicans* Zcf21, so we could not perform an independent check of its motif. Taken together, the fact that we were able to derive motifs de novo from the ChIP data, and the similarity of these independently derived motifs to the sequences known to be bound by homologs in other species validate the dataset that we generated by genome-wide ChIP.

### Identification of Target Genes Reveals Interplay among Regulators of Intestinal Colonization and Systemic Infection

**Figure 6:**
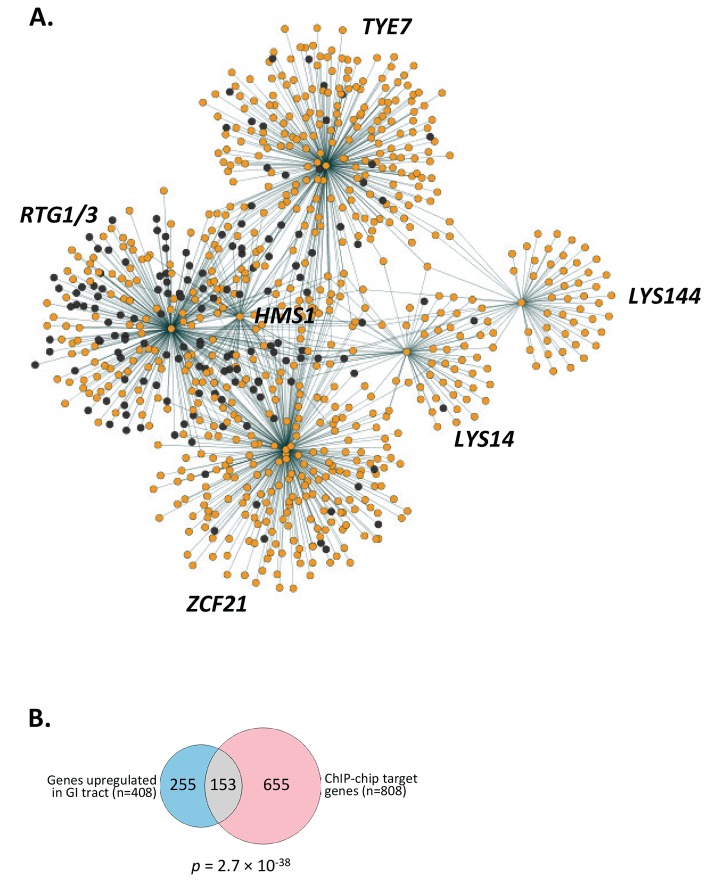
A gene regulatory network comprising *C. albicans* genes upregulated in the host. (A) Gene regulatory network depicting the established 808 target genes (orange circles) connected to their respective TRs (hubs) by dashed lines which indicate a direct interaction as determined by ChIP-chip. Dark grey circles correspond to the 153 genes upregulated in the GI tract. (B) A significant proportion of the target genes identified by ChIP-chip (*n* = 808) corresponds to genes upregulated when *C. albicans* grows in the GI tract (*n* = 408) [19]. The hypergeometric distribution was used to evaluate the significance of the overlap and its *p*-value is indicated.

All the binding events by the seven TRs (including the Tye7 ChIP data from [24]) translate into 808 putative target genes bound by at least one of the regulators (binding events in intergenic regions between divergently transcribed genes were counted as two target genes) (Dataset S2). The resulting network depicting the relationships among the regulators and all their target genes is shown in Figure 6A. It is apparent from the network's topology that many of the target genes are regulated by more than one TR. Moreover, the network displays no clear distinction between potential subsets of targets controlled specifically by regulators required for intestinal colonization and subsets controlled solely by regulators of systemic infection. In fact, there is no obvious partition among the sets of targets controlled by *RTG1*/*3*, *HMS1*, *TYE7*, and *ZCF21* even though the phenotypes ascribed to them are different: *RTG1*/*3* and *HMS1* were identified in both screens, *TYE7* only in the GI tract model, and *ZCF21* only in the systemic model.

### Rtg1/3 Is a Major Regulator of Genes Preferentially Expressed during Intestinal Colonization

This study was designed to identify TRs that specifically control aspects of *C. albicans* that are needed in the host. A prediction of this idea is that the target genes identified in this study will be preferentially expressed when *C. albicans* is in the host. To test this prediction, we compared the list of ChIP targets that we identified to an independently generated gene expression dataset where *C. albicans* growing in the mouse intestine was compared to *C. albicans* growing under laboratory conditions. In this study [19], Rosenbach et al. defined a collection of 408 genes that were upregulated during growth in the murine cecum relative to laboratory grown exponential and post-exponential phase cells (in reference [19]'s table S3). We found that *C. albicans* genes upregulated during growth in the mouse intestine are significantly overrepresented in the set of putative ChIP targets (153 out of 408 genes, *p* = 2.7×10^−38^) (Figure 6B; Dataset S2) supporting a role for the identified regulators in controlling a gene expression program activated specifically in the host.

The subset of 153 target genes upregulated when *C. albicans* is growing in the murine gut is not evenly distributed across the network (Figure 6A). Rather they are predominantly located in the set controlled by Rtg1/3 (108 of 153 genes) (Dataset S2) suggesting that these two proteins are major regulators of GI tract colonization determinants. *RTG1*/*3* controls mitochondrial retrograde signaling in *S. cerevisiae* (reviewed in [29]). This pathway involves sensing and transmitting nutritional as well as mitochondrial signals to effect changes in nuclear gene expression; these changes lead to a reconfiguration of metabolism to accommodate cells to nutrient availability or to mitochondrial defects [29]. Based on our ChIP-chip results, Rtg1/3 appears to regulate similar functions in *C. albicans* and these functions seem to contribute to the ability of the fungus to proliferate in the GI tract. In support of this idea, the subset of Rtg1/3 targets upregulated in *C. albicans* cells growing in the intestine (108 genes) is enriched with genes involved in metabolic functions (e.g., carbohydrate catabolic process [*p* = 3.28×10^−5^]).

### Genes Encoding Putative Amino Acid Permeases and Allantoate Transporters Are Targets of the Regulators Required for GI Tract Colonization

While there is a diverse set of biological functions and processes represented in the target genes in the identified network (Figure 6A), two groups of membrane proteins are salient among the targets bound by the TRs required for intestinal colonization: First, about a third of the *C. albicans* genes annotated as encoding amino acid permeases (*GNP1*, *HIP1*, *CAN2*, *AGP2*, *GAP2*, and *GAP6*) are bound by Rtg1/3. Moreover, Rtg1/3 and Hms1 bind upstream of *STP2*, a gene encoding a major regulator of transcription of amino acid permeases in *C. albicans* [32]. And second, Lys144 binds upstream of each of four putative allantoate transporters (*DAL5*, *DAL7*, *DAL8*, and *DAL9*) and of *ORF19.2065*, a gene whose ortholog in *S. cerevisiae* (*DAL2*) encodes an enzyme involved in allantoate catabolism [33]. That these TRs may exert control on the acquisition of amino acids as well as of allantoate, a product of purine metabolism in some species, suggest that, in the gut, *C. albicans* adjusts its metabolic response to procure nitrogen from these molecules.

### Target Genes Implicated in Intestinal Colonization and Systemic Infection

We next wanted to test experimentally whether the target genes identified in this study were actually required for *C. albicans* to colonize the GI tract or for fitness during systemic infection. We reasoned that the most likely candidates to show strong effects would be those genes that are clearly bound by one or more of the TRs identified here and whose expression is upregulated when *C. albicans* is growing in the mouse compared to laboratory conditions. Of the 153 genes upregulated in the host (Figure 6), we focused on those bound by Hms1 and Rtg1/3 because these TRs showed phenotypes in both mouse models. We selected 18 genes that met these criteria (Table S2) and successfully constructed signature-tagged homozygous deletion strains for 17 of these genes (we were unable to make a homozygous deletion of *orf19.1363*, raising the possibility that this gene may be essential) and tested 15 of them in the mouse models of GI tract colonization and systemic infection (*orf19.1069* and *orf19.4961* were excluded because their deletion results in severe growth defects in vitro). As described for the initial TR screen, we tested these mutants as a single pool in at least six mice.

**Figure 7:**
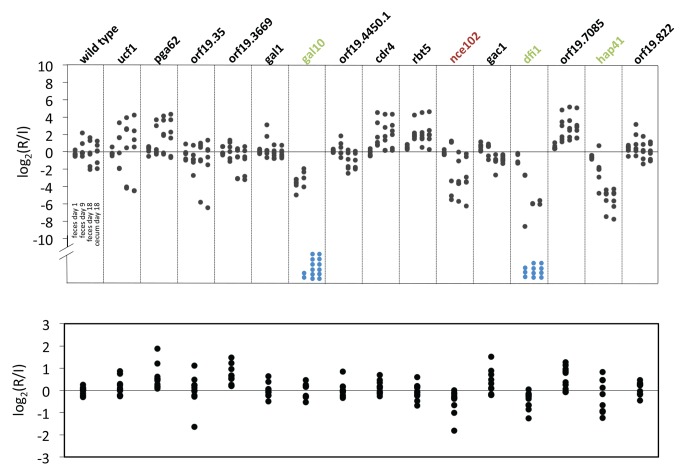
Target genes required for proliferation in the host. GI tract colonization (top) and systemic infection (bottom) analyses of selected target genes upregulated in the GI tract and controlled by both *HMS1* and *RTG1/3*. Blue dots represent samples below the qPCR detection level. Mutants with statistically significant GI tract colonization impairment are in green; statistically significant systemic infection defect is indicated in red.

Three of the 15 homozygous deletion mutants (*gal10* [*orf19.3672*], *dfi1* [*orf19.7084*], and *hap41* [*orf19.740*]) showed significantly reduced levels of GI tract colonization whereas one (*nce102* [*orf19.5960*]) displayed reduced fungal burden in kidneys after tail vein infection (Figure 7). (Although *dfi1* did not meet statistical significance, it shows a trend towards reduced fungal burden, which is consistent with a previous report [34]). As predicted by the ChIP-chip–based network (Figure 6A), Rtg1/3 and Hms1 regulate the expression levels of these targets (Figure S2). None of the identified genes had been previously implicated in intestinal colonization. While the *C. albicans GAL10* gene encodes an enzyme of the galactose utilization pathway [35], the ability to use galactose as a carbon source *per se* is unlikely to contribute to the mutant's inability to colonize the GI tract because we did not observe similar defects in the *GAL1* mutant (Figure 7) (the *GAL1* gene encodes another enzyme of the galactose utilization pathway). Rather, the colonization defect may be related to the anomalous cell wall ultrastructure found in the *gal10* mutant, an observation supported by the increased sensitivity of the mutant to cell wall disturbing agents such as Congo Red [35]. *DFI1* encodes a cell wall-linked protein that promotes invasive filamentation when *C. albicans* is grown in semi-solid medium [34]. Like the *gal10* mutant, the *C. albicans dfi1* mutant is hypersensitive to cell wall disturbing agents such as Congo Red and Caspofungin [34]. This observation implicates determinants of cell surface integrity in the ability of *C. albicans* to colonize the murine GI tract. *DFI1* also appears to signal through the Cek1 kinase to promote adhesion in addition to filamentation [34]; both properties seem important for the fungus to endure in the intestine.

Little is known about the function of *HAP41* and *NCE102* in *C. albicans*. *HAP41* is a *S. cerevisiae HAP4* homolog but lacks a DNA-binding domain. In *S. cerevisiae*, the heme-activated, glucose-repressed Hap2p/3p/4p/5p CCAAT-binding complex is a transcription activator and global regulator of respiratory gene expression. The *C. albicans* genome harbors multiple homologs of each of the subunits of the *S. cerevisiae* complex. Unlike other *HAP* gene transcripts, *HAP41* does not respond to iron deprivation conditions in *C. albicans* [36] suggesting that its function may be different from its *S. cerevisiae* homologs. The *S. cerevisiae NCE102* gene encodes a transmembrane protein localized to discrete membrane compartments [37] and has been implicated in protein export [38] and as a sensor of sphingolipids [37]. To our knowledge, this is the first report that *C. albicans NCE102* plays a role in the host.

## Discussion

**Figure 8:**
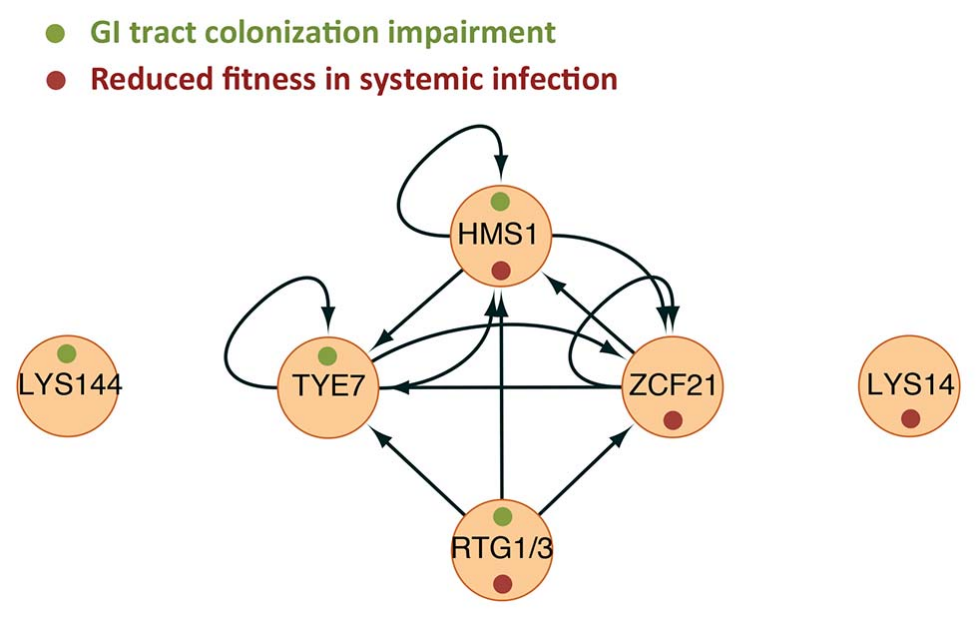
Interplay among *C. albicans* transcription regulators controlling proliferation in the host. The ChIP-chip–based interactions among the identified TRs are shown. Arrows indicate direct binding events. Green and red dots depict the phenotype associated with each TR.

We have investigated the transcriptional regulatory circuits and the repertoire of genes that the opportunistic pathogen *C. albicans* uses to thrive in two niches within its mammalian host. The identification of TRs that play roles predominantly during GI tract colonization (*TYE7*, *ORF19.3625*, and *LYS144*) or during systemic infection (*ZCF21* and *LYS14*) as well as of TRs required in both locales (*RTG1*/*3* and *HMS1*) suggest that these two disparate niches impose both exclusive and shared demands upon *C. albicans*. Our finding that some TRs show strong defects in only one of the two mouse models is similar to what has been reported for a *tec1* mutant strain and for a strain ectopically expressing *EFH1* [19],[20],[39]. However, the high degree of interconnectedness that we observe among the identified TRs (Figure 8) and in the entire gene network (Figure 6A) indicate that these are not circuits dedicated exclusively to one or the other niche. Rather, our results indicate that a large interconnected network functions in both niches and that the expression of target genes in one locale or the other is coordinated by this network.

Our finding that a shared regulatory network controls aspects of both commensalism (i.e., GI tract colonization) and systemic infection (i.e., fungal burden in kidneys after bloodstream infection) may be rationalized in the context of the natural history of *C. albicans*: while its association with mammals may be ancient [6], the selective pressure on the fungus has likely been as a commensal organism. In fact, *Candida* spp. were considered essentially non-pathogenic until the mid-1950s [40]. Thus, the functions that confer on *C. albicans* the ability to produce systemic infections are likely built upon the available regulatory circuitry that allows *C. albicans* to proliferate in its host as a commensal organism.

We focused our genetic screens on a subset (∼35%) of the TRs present in the genome of *C. albicans*. Essential TRs as well as regulator mutants that display moderate to strong in vitro phenotypes were excluded from our screen because we wanted to identify genes explicitly needed for *C. albicans* to colonize different niches of mammalian hosts. TRs not included in our screen, however, can also contribute to the proliferation of the fungus in the host. For example, *CPH2*, a regulator of hyphal development in *C. albicans* [41], has been shown to be required to colonize the murine GI tract [19]; a similar phenotype in the mouse is observed when *EFH1*, a regulator of pseudohyphal formation [42], is overexpressed [20]. The TRs identified in our study as defective in gut colonization may control these two regulators because Rtg1/3 and Hms1 bind upstream of the *CPH2* gene whereas Tye7 binds upstream of the *EFH1* gene (Dataset S2). The inclusion in our network (Figure 6A) of the two regulators previously known to affect gut colonization suggests that a significant proportion of the “gene clusters” that contribute to the growth of *C. albicans* in the host are linked with one another.

The topology of the gene network that results from our analysis (Figures 6A and 8) reveals that it contains a highly interconnected core component (composed of the TRs *RTG1*/*3*, *TYE7*, *ZCF21*, and *HMS1*) and a remote, “satellite-like” component (circuits controlled by *LYS14* and *LYS144*). Within the core component, *RTG1*/*3* appear to be the only “master regulators” that are not transcriptionally regulated by themselves or the other TRs (Figures 8 and S3). This may reflect the fact that Rtg1 and Rtg3 are regulated post-translationally (by phosphorylation and translocation into the nucleus) and not at the transcriptional level [29]. The core component resembles other highly interwoven circuits known to direct well-established cell differentiation processes such as white-opaque switching [43] and biofilm development [44] in *C. albicans* or filamentation in *S. cerevisiae* [45]. Our findings, therefore, support the notion that *C. albicans* employs an integrated regulatory circuit to control the expression of genes that allow it to thrive in the host.

The gene network that we have identified as controlling proliferation in the host is enriched with genes upregulated when *C. albicans* grows in the mouse intestine (Figure 6). The finding that this subset of 153 target genes is predominantly located around Rtg1/3 in the network (108 of 153 genes) suggests that these two proteins are major regulators of GI tract colonization. Among these determinants, the ability to regulate metabolic functions such as sugar catabolism appears to be particularly important for the fungus to successfully colonize the GI tract: the subset of 108 Rtg1/3 targets upregulated in *C. albicans* cells growing in the intestine is enriched with genes that play crucial roles in this process. Consistent with the notion that regulating metabolic functions is pivotal for intestinal colonization, sugar catabolism is a function enriched also among the targets of *HMS1* (hexose catabolic process [*p* = 2.7×10^−4^]), a regulator necessary for gut colonization as well (Figure 2). Based on our circuit mapping (Figures 6 and S4), the function of *RTG1*/*3* in *C. albicans* is similar, at least in broad outline, to that of their orthologs in *S. cerevisiae* where they control mitochondrial retrograde signaling (reviewed in [29]). This pathway involves sensing and transmitting nutritional as well as mitochondrial signals to effect changes in nuclear gene expression, which lead to a reconfiguration of metabolism to accommodate cells to nutrient availability or mitochondrial defects.

### Prominent Functions Controlled by Regulators of Intestinal Colonization and Systemic Infection

The collection of TR target genes in the network includes a large and diverse set of biological functions, but three broad functions/categories are most noticeable: (1) acquisition and metabolism of carbon; (2) acquisition and metabolism of nitrogen; and (3) transporters and cell surface proteins. The acquisition and metabolism of carbon and nitrogen are among the most prominent challenges faced by bacteria that live in the gut as well [46]. Moreover, bacterial pathogens that undergo mutations as well as gene gains/losses resulting in alterations of their metabolic capabilities often display a selective advantage [47]. Cell surface remodeling is a key strategy used by microorganisms to circumvent host defenses; in fact, the ability to do so has been demonstrated to contribute to the virulence of a broad range of pathogens including bacteria [48], fungi [12],[49], and parasites [50]. In bacterial species that can turn from harmless commensals to life-threatening pathogens, surface proteins also appear to play major roles in the transition between commensalism and pathogenicity [51].

Carbohydrates consumed by the gut microbiota are typically oligo- or polysaccharides derived from diet, host mucosal secretion, or other resident (or dietary) microbes [46]. In the bloodstream, on the other hand, glucose is the only sugar available whereas in internal organs the carbohydrates available are probably those from the proteoglycans that form the extracellular matrix, an ubiquitous constituent of animal tissues [52]. This major difference in the potential source of carbon between the two locales suggest that the strategy that *C. albicans* employs to obtain carbohydrates in the GI tract should differ, at least in part, from the strategy used while in the bloodstream or internal organs. Consistent with this notion, we find that *TYE7*, one of the major regulators of carbohydrate metabolism in *C. albicans* [24], is needed to proliferate in the gut but not during systemic infection. *RTG1*/*3* and *HMS1*, both required not only for gut colonization but also for full fitness after bloodstream infection, bind upstream of a significant number of genes involved in hexose catabolism (Dataset S2). This function may be important during systemic infection because genes involved in the assimilation of alternative carbon sources have been found to be upregulated in *C. albicans* cells during infection of the mammalian kidney [53] and carbon metabolism has been implicated in the infections of other fungal pathogens as well [54]. Metabolic flexibility, in general, has been postulated to be a requisite for *C. albicans* infection due to the dynamic nature of host niches which contain complex arrays of nutrients [55].

How nitrogen is acquired by microorganisms living in the GI tract remains an open question. Several bacterial species that live in the gut, e.g., *Bacterioides*, seem to rely on NH_3_ (reviewed in [46]). Other sources could be amino sugars and proteins that are present in secreted mucus and epithelial cells, or amino acids derived from diet [46]. Consistent with the latter, Rtg1/3, one of the *C. albicans* TRs controlling intestinal colonization, has a number of putative amino acid permeases among their target genes. In addition, we find that Lys144 binds upstream of each of four putative allantoate transporters raising the possibility that allantoate, a product of purine catabolism in some bacteria, is one of the sources of nitrogen for *C. albicans* in the gut. Contrary to what their nomenclature implies, neither Lys144 nor Lys14 seems to regulate lysine biosynthesis genes in *C. albicans* (our ChIP data and phenotypic screen results in [16]).

The majority of the pathogen-associated molecular patterns (PAMPs) that activate and modulate immune responses are cell wall components [56],[57]. Indeed, *C. albicans* mutants that are unable to add particular carbohydrate moieties to their surface proteins are attenuated for virulence in mouse models of systemic infection. *ZCF21* and *LYS14*, the two TRs that influence the outcome of systemic infections but not colonization of the GI tract, have among their targets a significant number of genes encoding proteins predicted to be localized to the cell surface or enzymes that modify the cell wall structure such as the mannosyltransferase *OCH1* and the glucosyltransferase *ALG6*. Hence, our findings reveal two regulators that *C. albicans* employ to remodel its surface and indicate that these modifications are needed during systemic infection.

In summary, our findings indicate that the ability of *C. albicans* to colonize multiple niches within a mammalian host relies on a large, integrated circuit that responds to different environmental conditions to effect major changes in metabolic functions, nutrient (especially carbon and nitrogen) acquisition, cell wall remodeling, and cell wall integrity. We propose that this “master circuit” allows *C. albicans* to adjust to disparate environments in the host and accounts for the close links between commensalism and pathogenicity.

## Materials and Methods

Strains and primers used in this study are listed in Tables S3 and S4, respectively. All *C. albicans* strains were derived from the wild-type strain SN152 [58]. Gene deletions were constructed as described [58]; the *TDH3* promoter-driven overexpression strains were generated using the plasmids and procedures described in [59]; the strategies and protocols detailed in Hernday et al. [60] were used for MYC-, GFP-, and YFP-gene tagging. All procedures involving animals were approved by the UCSF Institutional Animal Care and Use Committee.

### Gastrointestinal Tract Colonization Model

The procedure used was essentially the one described [19],[20]. Female Swiss Webster mice (18–20 g) were treated with antibiotics (tetracycline [1 mg/ml]), streptomycin [2 mg/ml], and gentamycin [0.1 mg/ml]) added to their drinking water throughout the experiment beginning 4 d before inoculation. Prior to inoculation, *C. albicans* strains were grown for ∼18 h at 30°C in YPD liquid medium, washed twice with PBS, and counted in a hemocytometer. Mice were orally inoculated with 5×10^7^ *C. albicans* cells (in a 0.1 or 0.2 ml volume) by gavage using a feeding needle. Colonization was monitored by collecting fecal pellets (produced within 10 min prior to collection) at various days post-inoculation and cecum contents at the end of the experiment when the mice were killed. In the initial screening the fecal pellets and intestinal contents were used to prepare genomic DNA directly from the samples. In follow-up experiments, the mouse homogenates were plated onto Sabouraud medium containing ampicillin (50 µg/ml) and gentamycin (15 µg/ml) (antibiotics were included to prevent the growth of contaminating bacteria). Genomic DNA was prepared from yeast scraped off the plates. The yields of DNA prepared directly from fecal pellets and intestinal contents were relatively low, hence one round of whole genome amplification (using Sigma's GenomePlex Complete Whole Genome Amplification kit) was used to generate adequate amounts of material before the qPCR analysis. Similar results were obtained with samples that were plated or that were processed directly.

The 77 *C. albicans* deletion mutants screened are listed in Table S1. We assayed pools of 15–20 signature-tagged mutant strains. The relative abundance of the strains recovered from feces (at 1, 9, and 18 or 21 d post-inoculation) or intestinal contents (at day 18 or 21) compared with the inoculum was determined by real time PCR (using primers to the signature tags) as described [15]. Briefly, threshold cycle (*C*_T_) values were converted to a linear scale using the simple equation, linear value = 2^−*C*T^. Experiments comparing 15 strains resulted in 15 values for the inoculum (*I*) and another 15 for the recovered pool (*R*_raw_). *R*_raw_ values were multiplied by median(*I*)/median(*R*_raw_) to generate normalized *R* values. Ultimately, *R*/*I* was calculated for each mutant strain. These ratios expressed as log_2_ values are shown in Figure 2A.

Empirically we found that the limit of accurate qPCR detection for most strains was about 1,000-fold reduction in levels compared to the inoculum. This level of detection is consistent with the number of colonies, typically ∼10,000, that we recovered after plating around 10 mg of the fecal pellet and intestinal content homogenates.

### Systemic Infection Model

The procedure used has been described by our laboratory [15]. We used the *t*-test to compare the log_2_(*R*/*I*) of mutants to those of wild-type using a significance threshold of *p*<0.05 (correcting for multiple comparisons).

### Virulence Analysis of Single Infections

Ten female BALB/c mice (18–20 g) were infected with wild-type *C. albicans* or one of the mutant strains by tail vein injection. Saturated *C. albicans* cultures were diluted 1∶25 in YPD and grown for ∼4 h at 30°C prior to infection. Cells were washed twice with sterile saline, counted in a hemocytometer and 5.2×10^5^ cells (in a 0.1 ml volume) were injected in each mouse. Mice were monitored daily and sacrificed when moribund. The logrank test was used for statistical analysis

### Full-Genome Chromatin Immunoprecipitation

Each TR was tagged with a 13-MYC or GFP tag at the C- or N-terminal end of the protein in a wild-type reference strain background. The tagged strains along with untagged controls were grown as indicated in Figure 5 and ChIP was carried out as described [60] with the following modifications: GFP-tagged regulators were immunoprecipitated with an anti-rGFP polyclonal antibody (Clontech); the DNA recovered after crosslink reversal was purified with QIAquick PCR purification columns (Qiagen) and amplified using the GenomePlex Complete Whole Genome Amplification kit (Sigma). Input and immunoprecipitated DNA were fluorescently labeled and competitively hybridized to custom full-genome oligonucleotide tiling microarrays (Agilent) as described [44]. MochiView [61] was used for data visualization, identification of binding events, and DNA motif analysis.

### ChIP-Chip Data Analysis

The microarray data were normalized using the global lowess method. The normalized log_2_ enrichment values (IP/input) for each probe were imported into MochiView and the software's default parameters were used to smooth the data and extract binding events (peaks). The cutoff for the minimum value for peak inclusion was set at two or three standard deviations from the mean of the log_2_ enrichment values (cutoffs were typically in the range of 0.6–0.8). To ensure the generation of a high confidence dataset, in addition to the standard analysis performed by MochiView we manually curated all the extracted peaks using the following criteria: (1) ChIP data derived from untagged control strains immunoprecipitated with anti-MYC and anti-GFP antibodies were used to filter out non-specific peaks (this function is incorporated in MochiView); (2) peaks located within annotated ORFs were discarded; (3) peaks located around highly expressed genes (particularly ribosomal genes) were also discarded because based on our experience (e.g., [43],[44],[62]) these places tend to bind to almost all DNA-binding proteins non-specifically; and (4) we only included peaks that were consistent in two independent biological replicates (typically >80% of peaks were concordant in the replicates).

### DNA Motif Analysis

Sequences of 500 nt centered on the midpoint of about 20–30 of the top-scoring peaks for each regulator were used to derive motifs in MochiView. The software's default parameters were employed. To assess the significance of the derived motifs, we compared their occurrence in the remaining peaks versus their occurrence in a set of random intergenic regions of the same length. This analysis was performed using MochiView's “enrichment” function.

### Statistical Analysis

The logrank test was used to compare the persistence or depletion of the various *C. albicans* mutants in the murine GI tract (Figure 2B) and to compare the time-to-illness curves of monotypic infections (Figure 4B–4D). The *t*-test (two-tailed, comparison of unpaired samples) was used to evaluate the significance of the log_2_ (*R*/*I*) values of the mutants versus the wild-type reference strain in the systemic infection screen (correcting for multiple comparisons). The hypergeometric distribution was used to evaluate the significance of the overlap between sets of genes. The Gene Ontology Term Finder feature of the Candida Genome Database (www.candidagenome.org) was used to search biological processes or functions enriched in the various datasets.

### Gel Mobility Shift Assays

EMSAs were carried out as described previously [63].

### RNA Purification, Reverse Transcription-PCR, and Real-Time PCR to Quantify Transcript Levels

Cells were grown to mid-late logarithmic phase in YPD or Todd Hewitt Broth at 30°C. Total RNA was prepared with the RiboPure-Yeast kit (Ambion, Life Technologies) following the manufacturer's instructions. Three micrograms of purified RNA per sample were used to synthesize cDNA with SuperScript II Reverse Transcriptase (Invitrogen). Quantification of transcripts was performed by real-time PCR using SYBR green. Results were normalized to those of the actin gene (*ACT1*).

### Accession Number

The ChIP-chip data reported in this article have been deposited in the NCBI Gene Expression Omnibus (GEO) database under accession number GSE41237.

## Supporting Information

Dataset S1 ChIP-chip binding regions and log_2_ enrichment values for Rtg1, Rtg3, Hms1, Lys14, Lys144, and Zcf21. The chromosome coordinates given follow the *C. albicans* SC5314 Assembly 19 (www.candidagenome.org) and are centered on the midpoint of each ChIP peak and extend 250 nt to each side. The ntar (novel transcriptionally active region) nomenclature is based on reference [44] of the main text.
(ZIP)

Dataset S2 List of target genes (ORFs) based on ChIP-chip data for Rtg1, Rtg3, Hms1, Lys14, Lys144, Zcf21, and Tye7.
(ZIP)

Figure S1 **DNA motifs derived from the ChIP-chip analysis occur preferentially in regions bound by the regulators.** The frequency with which each DNA motif occurs in the entire set of ChIP peaks (500-nt sequences centered in the midpoint of the peak) for a given regulator (blue line) versus in an equally sized set of random intergenic regions (red line) was evaluated in MochiView using its “enrichment plot” function. In all the plots shown, the blue line runs to the right of the red line indicating that at any motif score that one chooses as a cutoff, the set of ChIP peaks contains a higher proportion of matches to the motif than the set of random sequences. The Lys14 motif is composed of CGC repeats separated by 4 or 5 nt; since a fixed-length motif is required to perform the “enrichment” analysis, two plots are shown for this regulator.
(EPS)

Figure S2 ***HMS1*** **and** ***RTG1*** **/** ***3*** **control the expression of target genes that display impaired murine GI tract colonization.** *GAL10*, *DFI1*, *HAP41*, and *NCE102* expression levels in *C. albicans* wild-type, *hms1*, and *rtg1* deletion mutant strains. Transcript levels were determined by quantitative real-time PCR and normalized to *ACT1* levels. The levels of the transcripts in the wild-type strain are set to one to facilitate comparisons. Shown are the means and standard deviation of two independent experiments performed in duplicates.
(EPS)

Figure S3 **Deletion and overexpression of the various transcription regulators composing the core circuit cause alterations in the expression of the other components.** *HMS1*, *ZCF21*, *TYE7*, and *RTG1* expression levels in *C. albicans* wild-type, *hms1*, *rtg1*, *zcf21*, and *tye7* deletion mutant strains, and in *HMS1*, *ZCF21*, *TYE7*, and *RTG1* overexpression strains. Transcript levels were determined by quantitative real-time PCR and normalized to *ACT1* levels. The levels of the transcripts in the wild-type strain are set to one to facilitate comparisons. Shown are the means and standard deviation of two independent experiments performed in duplicates.
(EPS)

Figure S4 ***RTG1*** **/** ***3*** **control the expression of genes that have metabolic functions.** Shown are the genes differentially regulated (log_2_>1 and log_2_<1) in gene expression array experiments that compared *rtg1* and *rtg3* deletion mutants to the wild-type reference strain. The order in which the genes are displayed reflect hierarchical clustering. Predicted gene functions are included if such information was available in the Candida Genome Database. About two-thirds of the genes with ascribed functions play metabolism-related roles. Cell culture and RNA purification were carried out as described under Materials and Methods. The procedures used for cDNA synthesis and labeling, array hybridization, data acquisition, and processing followed those described in reference [44] of the main text. Shown are the results of two biological replicates. A red dot indicates that Rtg1/3 bind upstream of the gene as determined by ChIP-chip.
(EPS)

Table S1 Fitness of TR mutant strains in mouse model of disseminated candidiasis. The log_2_ (recovered/input) values for each mutant in every mouse are shown.
(PDF)

Table S2 Target genes selected for testing role in gut colonization and systemic infection.
(PDF)

Table S3 Strains used in this study.
(PDF)

Table S4 Primers used in this study.
(PDF)

## Abbreviations

ChIP: chromatin immunoprecipitation
GI: gastrointestinal
TR: transcription regulator

## References

[1] Iliev ID, Funari VA, Taylor KD, Nguyen Q, Reyes CN, et al (2012) Interactions between commensal fungi and the C-type lectin receptor Dectin-1 influence colitis. Science 336: 1314–1317. 22674328 10.1126/science.1221789 PMC3432565

[2] Qin J, Li R, Raes J, Arumugam M, Burgdorf KS, et al (2010) A human gut microbial gene catalogue established by metagenomic sequencing. Nature 464: 59–65. 20203603 10.1038/nature08821 PMC3779803

[3] Walter J, Ley R (2011) The human gut microbiome: ecology and recent evolutionary changes. Annu Rev Microbiol 65: 411–429. 21682646 10.1146/annurev-micro-090110-102830

[4] Ghannoum MA, Jurevic RJ, Mukherjee PK, Cui F, Sikaroodi M, et al (2010) Characterization of the oral fungal microbiome (mycobiome) in healthy individuals. PLoS Pathog 6: e1000713 doi:10.1371/journal.ppat.1000713. 20072605 10.1371/journal.ppat.1000713 PMC2795202

[5] Odds FC (1987) *Candida* infections: an overview. Crit Rev Microbiol 15: 1–5. 10.3109/10408418709104444 3319417

[6] Odds FC (1988) *Candida* and candidosis. London: Bailliere Tindall.

[7] Calderone RA (2002) *Candida* and candidiasis. Washington (D.C.): ASM Press.

[8] Koh AY, Kohler JR, Coggshall KT, Van Rooijen N, Pier GB (2008) Mucosal damage and neutropenia are required for *Candida albicans* dissemination. PLoS Pathog 4: e35 doi:10.1371/journal.ppat.0020035. 18282097 10.1371/journal.ppat.0040035 PMC2242836

[9] Romani L (1999) Immunity to *Candida albicans*: Th1, Th2 cells and beyond. Curr Opin Microbiol 2: 363–367. 10458979 10.1016/S1369-5274(99)80064-2

[10] Calderone RA, Gow NA (2002) Host recognition by *Candida* species. Calderone RA, editor. *Candida* and Candidiasis. Washington (D.C.): ASM Press. pp. 67–86.

[11] Hube B, Naglik JR (2002) Extracellular Hydrolases. Calderone RA, editor. *Candida* and Candidiasis. Washington (D.C.): ASM Press. pp. 107–122.

[12] Gow NA, van de Veerdonk FL, Brown AJ, Netea MG (2012) *Candida albicans* morphogenesis and host defence: discriminating invasion from colonization. Nat Rev Microbiol 10: 112–122. 10.1038/nrmicro2711 PMC3624162 22158429

[13] Lo HJ, Kohler JR, DiDomenico B, Loebenberg D, Cacciapuoti A, et al (1997) Nonfilamentous *C. albicans* mutants are avirulent. Cell 90: 939–949. 9298905 10.1016/s0092-8674(00)80358-x

[14] Brunke S, Hube B (2013) Two unlike cousins: *Candida albicans* and *C. glabrata* infection strategies. Cell Microbiol In press. 10.1111/cmi.12091 PMC3654559 23253282

[15] Noble SM, French S, Kohn LA, Chen V, Johnson AD (2010) Systematic screens of a *Candida albicans* homozygous deletion library decouple morphogenetic switching and pathogenicity. Nat Genet 42: 590–598. 20543849 10.1038/ng.605 PMC2893244

[16] Homann OR, Dea J, Noble SM, Johnson AD (2009) A phenotypic profile of the *Candida albicans* regulatory network. PLoS Genet 5: e1000783 doi:10.1371/journal.pgen.1000783. 20041210 10.1371/journal.pgen.1000783 PMC2790342

[17] Liu OW, Chun CD, Chow ED, Chen C, Madhani HD, et al (2008) Systematic genetic analysis of virulence in the human fungal pathogen *Cryptococcus neoformans* . Cell 135: 174–188. 18854164 10.1016/j.cell.2008.07.046 PMC2628477

[18] Shea JE, Santangelo JD, Feldman RG (2000) Signature-tagged mutagenesis in the identification of virulence genes in pathogens. Curr Opin Microbiol 3: 451–458. 11050441 10.1016/s1369-5274(00)00120-x

[19] Rosenbach A, Dignard D, Pierce JV, Whiteway M, Kumamoto CA (2010) Adaptations of *Candida albicans* for growth in the mammalian intestinal tract. Eukaryot Cell 9: 1075–1086. 20435697 10.1128/EC.00034-10 PMC2901676

[20] White SJ, Rosenbach A, Lephart P, Nguyen D, Benjamin A, et al (2007) Self-regulation of *Candida albicans* population size during GI colonization. PLoS Pathog 3: e184 doi:10.1371/journal.ppat.0030184. 18069889 10.1371/journal.ppat.0030184 PMC2134954

[21] Chen C, Pande K, French SD, Tuch BB, Noble SM (2011) An iron homeostasis regulatory circuit with reciprocal roles in *Candida albicans* commensalism and pathogenesis. Cell Host Microbe 10: 118–135. 21843869 10.1016/j.chom.2011.07.005 PMC3165008

[22] Pierce JV, Dignard D, Whiteway M, Kumamoto CA (2013) Normal adaptation of *Candida albicans* to the murine GI tract requires Efg1p-dependent regulation of metabolic and host defense genes. Eukaryot Cell 12: 37–49. 23125349 10.1128/EC.00236-12 PMC3535844

[23] Pierce JV, Kumamoto CA (2012) Variation in *Candida albicans EFG1* expression enables host-dependent changes in colonizing fungal populations. mBio 3: e00117–12. 22829676 10.1128/mBio.00117-12 PMC3413400

[24] Askew C, Sellam A, Epp E, Hogues H, Mullick A, et al (2009) Transcriptional regulation of carbohydrate metabolism in the human pathogen *Candida albicans* . PLoS Pathog 5: e1000612 doi:10.1371/journal.ppat.1000612. 19816560 10.1371/journal.ppat.1000612 PMC2749448

[25] Bonhomme J, Chauvel M, Goyard S, Roux P, Rossignol T, et al (2011) Contribution of the glycolytic flux and hypoxia adaptation to efficient biofilm formation by *Candida albicans* . Mol Microbiol 80: 995–1013. 21414038 10.1111/j.1365-2958.2011.07626.x

[26] Shapiro RS, Sellam A, Tebbji F, Whiteway M, Nantel A, et al (2012) Pho85, Pcl1, and Hms1 signaling governs *Candida albicans* morphogenesis induced by high temperature or Hsp90 compromise. Curr Biol 22: 461–470. 22365851 10.1016/j.cub.2012.01.062

[27] Jia Y, Rothermel B, Thornton J, Butow RA (1997) A basic helix-loop-helix-leucine zipper transcription complex in yeast functions in a signaling pathway from mitochondria to the nucleus. Mol Cell Biol 17: 1110–1117. 9032238 10.1128/mcb.17.3.1110 PMC231836

[28] Liao X, Butow RA (1993) *RTG1* and *RTG2*: two yeast genes required for a novel path of communication from mitochondria to the nucleus. Cell 72: 61–71. 8422683 10.1016/0092-8674(93)90050-z

[29] Liu Z, Butow RA (2006) Mitochondrial retrograde signaling. Annu Rev Genet 40: 159–185. 16771627 10.1146/annurev.genet.40.110405.090613

[30] Becker B, Feller A, el Alami M, Dubois E, Pierard A (1998) A nonameric core sequence is required upstream of the LYS genes of *Saccharomyces cerevisiae* for Lys14p-mediated activation and apparent repression by lysine. Mol Microbiol 29: 151–163. 9701810 10.1046/j.1365-2958.1998.00916.x

[31] Kim JB, Spotts GD, Halvorsen YD, Shih HM, Ellenberger T, et al (1995) Dual DNA binding specificity of ADD1/SREBP1 controlled by a single amino acid in the basic helix-loop-helix domain. Mol Cell Biol 15: 2582–2588. 7739539 10.1128/mcb.15.5.2582 PMC230488

[32] Martinez P, Ljungdahl PO (2005) Divergence of Stp1 and Stp2 transcription factors in *Candida albicans* places virulence factors required for proper nutrient acquisition under amino acid control. Mol Cell Biol 25: 9435–9446. 16227594 10.1128/MCB.25.21.9435-9446.2005 PMC1265835

[33] Yoo HS, Genbauffe FS, Cooper TG (1985) Identification of the ureidoglycolate hydrolase gene in the *DAL* gene cluster of *Saccharomyces cerevisiae* . Mol Cell Biol 5: 2279–2288. 3915539 10.1128/mcb.5.9.2279-2288.1985 PMC366954

[34] Zucchi PC, Davis TR, Kumamoto CA (2010) A *Candida albicans* cell wall-linked protein promotes invasive filamentation into semi-solid medium. Mol Microbiol 76: 733–748. 20384695 10.1111/j.1365-2958.2010.07137.x PMC3163599

[35] Singh V, Satheesh SV, Raghavendra ML, Sadhale PP (2007) The key enzyme in galactose metabolism, UDP-galactose-4-epimerase, affects cell-wall integrity and morphology in *Candida albicans* even in the absence of galactose. Fungal Genet Biol 44: 563–574. 17178245 10.1016/j.fgb.2006.11.006

[36] Singh RP, Prasad HK, Sinha I, Agarwal N, Natarajan K (2011) Cap2-HAP complex is a critical transcriptional regulator that has dual but contrasting roles in regulation of iron homeostasis in *Candida albicans* . J Biol Chem 286: 25154–25170. 21592964 10.1074/jbc.M111.233569 PMC3137088

[37] Frohlich F, Moreira K, Aguilar PS, Hubner NC, Mann M, et al (2009) A genome-wide screen for genes affecting eisosomes reveals Nce102 function in sphingolipid signaling. J Cell Biol 185: 1227–1242. 19564405 10.1083/jcb.200811081 PMC2712959

[38] Cleves AE, Cooper DN, Barondes SH, Kelly RB (1996) A new pathway for protein export in *Saccharomyces cerevisiae* . J Cell Biol 133: 1017–1026. 8655575 10.1083/jcb.133.5.1017 PMC2120850

[39] Schweizer A, Rupp S, Taylor BN, Rollinghoff M, Schroppel K (2000) The TEA/ATTS transcription factor CaTec1p regulates hyphal development and virulence in *Candida albicans* . Mol Microbiol 38: 435–445. 11069668 10.1046/j.1365-2958.2000.02132.x

[40] Casadevall A, Pirofski LA (2003) The damage-response framework of microbial pathogenesis. Nat Rev Microbiol 1: 17–24. 15040176 10.1038/nrmicro732 PMC7097162

[41] Lane S, Zhou S, Pan T, Dai Q, Liu H (2001) The basic helix-loop-helix transcription factor Cph2 regulates hyphal development in *Candida albicans* partly via *TEC1* . Mol Cell Biol 21: 6418–6428. 11533231 10.1128/MCB.21.19.6418-6428.2001 PMC99789

[42] Doedt T, Krishnamurthy S, Bockmuhl DP, Tebarth B, Stempel C, et al (2004) APSES proteins regulate morphogenesis and metabolism in *Candida albicans* . Mol Biol Cell 15: 3167–3180. 15218092 10.1091/10.1091/mbc.E03-11-0782 PMC452574

[43] Zordan RE, Miller MG, Galgoczy DJ, Tuch BB, Johnson AD (2007) Interlocking transcriptional feedback loops control white-opaque switching in *Candida albicans* . PLoS Biol 5: e256 doi:10.1371/journal.pbio.0050256. 17880264 10.1371/journal.pbio.0050256 PMC1976629

[44] Nobile CJ, Fox EP, Nett JE, Sorrells TR, Mitrovich QM, et al (2012) A recently evolved transcriptional network controls biofilm development in *Candida albicans* . Cell 148: 126–138. 22265407 10.1016/j.cell.2011.10.048 PMC3266547

[45] Borneman AR, Leigh-Bell JA, Yu H, Bertone P, Gerstein M, et al (2006) Target hub proteins serve as master regulators of development in yeast. Gene Dev 20: 435–448. 16449570 10.1101/gad.1389306 PMC1369046

[46] Fischbach MA, Sonnenburg JL (2011) Eating for two: how metabolism establishes interspecies interactions in the gut. Cell Host Microbe 10: 336–347. 22018234 10.1016/j.chom.2011.10.002 PMC3225337

[47] Rohmer L, Hocquet D, Miller SI (2011) Are pathogenic bacteria just looking for food? Metabolism and microbial pathogenesis. Trends Microbiol 19: 341–348. 21600774 10.1016/j.tim.2011.04.003 PMC3130110

[48] Haraga A, Ohlson MB, Miller SI (2008) *Salmonellae* interplay with host cells. Nat Rev Microbiol 6: 53–66. 18026123 10.1038/nrmicro1788

[49] Gow NA, Hube B (2012) Importance of the *Candida albicans* cell wall during commensalism and infection. Curr Opin Microbiol 15: 406–412. 22609181 10.1016/j.mib.2012.04.005

[50] Cross GA (1996) Antigenic variation in trypanosomes: secrets surface slowly. BioEssays 18: 283–291. 8967896 10.1002/bies.950180406

[51] Wei W, Cao Z, Zhu YL, Wang X, Ding G, et al (2006) Conserved genes in a path from commensalism to pathogenicity: comparative phylogenetic profiles of *Staphylococcus epidermidis* RP62A and ATCC12228. BMC Genomics 7: 112. 16684363 10.1186/1471-2164-7-112 PMC1482698

[52] Chagnot C, Listrat A, Astruc T, Desvaux M (2012) Bacterial adhesion to animal tissues: protein determinants for recognition of extracellular matrix components. Cell Microbiol 14: 1687–1696. 22882798 10.1111/cmi.12002

[53] Walker LA, Maccallum DM, Bertram G, Gow NA, Odds FC, et al (2009) Genome-wide analysis of *Candida albicans* gene expression patterns during infection of the mammalian kidney. Fungal Genet Biol 46: 210–219. 19032986 10.1016/j.fgb.2008.10.012 PMC2698078

[54] Cairns T, Minuzzi F, Bignell E (2010) The host-infecting fungal transcriptome. FEMS Microbiol Lett 307: 1–11. 20557573 10.1111/j.1574-6968.2010.01961.x

[55] Sandai D, Yin Z, Selway L, Stead D, Walker J, et al (2012) The Evolutionary Rewiring of Ubiquitination Targets Has Reprogrammed the Regulation of Carbon Assimilation in the Pathogenic Yeast *Candida albicans* . mBio 3: e00495–12. 23232717 10.1128/mBio.00495-12 PMC3520108

[56] Netea MG, Brown GD, Kullberg BJ, Gow NA (2008) An integrated model of the recognition of *Candida albicans* by the innate immune system. Nat Rev Microbiol 6: 67–78. 18079743 10.1038/nrmicro1815

[57] Romani L (2011) Immunity to fungal infections. Nat Rev Immunol 11: 275–288. 21394104 10.1038/nri2939

[58] Noble SM, Johnson AD (2005) Strains and strategies for large-scale gene deletion studies of the diploid human fungal pathogen *Candida albicans* . Eukaryot Cell 4: 298–309. 15701792 10.1128/EC.4.2.298-309.2005 PMC549318

[59] Nobile CJ, Solis N, Myers CL, Fay AJ, Deneault JS, et al (2008) *Candida albicans* transcription factor Rim101 mediates pathogenic interactions through cell wall functions. Cell Microbiol 10: 2180–2196. 18627379 10.1111/j.1462-5822.2008.01198.x PMC2701370

[60] Hernday AD, Noble SM, Mitrovich QM, Johnson AD (2010) Genetics and molecular biology in *Candida albicans* . Methods Enzymol 470: 737–758. 20946834 10.1016/S0076-6879(10)70031-8

[61] Homann OR, Johnson AD (2010) MochiView: versatile software for genome browsing and DNA motif analysis. BMC Biol 8: 49. 20409324 10.1186/1741-7007-8-49 PMC2867778

[62] Tuch BB, Galgoczy DJ, Hernday AD, Li H, Johnson AD (2008) The evolution of combinatorial gene regulation in fungi. PLoS Biol 6: e38 oi:10.1371/journal.pbio.0060038. 18303948 10.1371/journal.pbio.0060038 PMC2253631

[63] Cain CW, Lohse MB, Homann OR, Sil A, Johnson AD (2012) A conserved transcriptional regulator governs fungal morphology in widely diverged species. Genetics 190: 511–521. 22095082 10.1534/genetics.111.134080 PMC3276625

