# Supplementary material for: Candida albicans Commensalism and Pathogenicity Are Intertwined Traits Directed by a Tightly Knit Transcriptional Regulatory Circuit
Source: PLoS Biol. 2013 Mar 19;11(3):e1001510. doi: 10.1371/journal.pbio.1001510 (PMC3601966; doi:10.1371/journal.pbio.1001510)
Supplement: Table S1 — Fitness of TR mutant strains in mouse model of disseminated candidiasis. The log2 (recovered/input) values for each mutant in every mouse are shown. (PDF) [file pbio.1001510.s007.pdf]

Table S1. Fitness of TR mutant strains in mouse model of disseminated candidiasis.

TR	log2(R/I) -->													median
orf19.921	-1.72	-1.35	-1.26	-1.92	-2.51	-2.14	-2.69	-1.36	-1.14	-2.33				**-1.82**
orf19.1499	-0.27	0.27	-0.47	0.51	-1.31	-3.24	-2.44	-4.04						**-0.89**
orf19.2646	0.00	1.59	0.69	0.61	-0.77	-1.37	-1.25							**0.00**
orf19.2647	0.17	0.16	0.35	-0.96	-1.51	-0.76	-1.57							**-0.76**
orf19.2730	0.06	-0.94	-1.20	-1.04	-2.70									**-1.04**
orf19.3190	0.80	0.26	0.67	-0.22										**0.46**
orf19.3305	1.21	0.74	0.96	1.33	-0.73	0.05	-1.23							**0.74**
orf19.3308	1.54	1.92	2.00	2.40	1.26									**1.92**
orf19.3434	2.66	2.34	2.38	2.35	-1.82	0.17	-0.64							**2.34**
orf19.3876	-0.43	-1.42	-1.33	-2.21	-2.46	1.48	1.75	2.61	3.02	2.92				**0.52**
orf19.3928	2.11	0.96	2.92	3.68	-0.33	-0.70	2.55							**2.11**
orf19.4145	0.74	-0.22	-0.12	-1.29										**-0.17**
orf19.4166	-3.11	-3.17	-3.13	0.13	-0.59	-0.92	-2.29	-2.58	-1.99	-1.47	-1.42	-1.39		**-1.73**
orf19.4225	-0.34	-0.87	-1.23	-0.45	-2.10									**-0.87**
orf19.4251	1.35	1.65	1.52	0.90	-2.77	-2.51	-4.01	-4.88						**-0.80**
orf19.4288	0.49	-0.06	0.71	0.33	-0.35	-0.24	0.38	0.83						**0.36**
orf19.4438	-0.11	0.48	0.96	0.38	2.84	2.67	4.29							**0.96**
orf19.4450	0.35	0.33	1.07	0.43	-1.15	-1.43	-1.32							**0.33**
orf19.4524	4.33	3.48	2.13	5.20	4.40									**4.33**
orf19.4568	1.22	1.68	0.76	1.18	1.58	1.09	0.92	0.61	1.16					**1.16**
orf19.4722	-2.39	-3.71	-4.38	-1.61	-1.70	-1.64	-1.65	-0.84	-2.91	-3.39	-2.20	-1.60	-2.10	**-2.10**
orf19.4778	0.96	1.01	0.04	-0.74	0.51	-1.82	-1.55	-0.96						**-0.35**
orf19.4941	-0.97	0.37	0.15	-0.93	0.24									**0.15**
orf19.4972	0.28	-0.25	0.18	-0.11										**0.03**
orf19.5001	0.33	0.11	0.76	0.38	0.86									**0.38**
orf19.5026	0.66	-0.07	0.73	0.35	-0.13									**0.35**
orf19.5097	0.51	0.01	1.14	2.28	0.50	0.34	0.15	0.09	1.38					**0.50**
orf19.5380	0.94	0.77	1.90	1.47	1.25	-0.86	-1.38	-0.97	-1.15	-1.77				**-0.04**
orf19.5498	-1.21	-0.77	-0.04	0.60	-2.87									**-0.77**
orf19.5548	-2.23	-1.01	-0.76	-0.38	-0.65	-1.13	-1.27	-1.92	-2.61	-1.14	-1.28	-2.25		**-1.21**
orf19.5651	0.99	0.65	0.87	0.63	0.42									**0.65**
orf19.5855	0.66	-0.58	-0.36	-1.75	-0.01	0.42	-0.16							**-0.16**
orf19.5940	0.28	-0.74	-1.55	-2.39	-0.67	-1.08	-0.41	-0.21						**-0.70**
orf19.5975	0.49	0.69	1.93	1.60	-1.68	-0.89	-0.71							**0.49**
orf19.6038	-3.61	-2.23	-1.54	-0.47	-1.36	-1.23	0.24	0.07	-0.81					**-1.23**
orf19.6514	-1.61	-1.54	-2.14	-1.93	-2.52	-0.23	0.37	0.33	-3.47					**-1.61**
orf19.7319	-0.93	-1.24	-2.08	-1.23	-1.80	-1.30	-1.77	-2.21	-0.82	-1.44				**-1.37**
orf19.7372	-0.43	0.16	-0.14	2.25	0.59	0.22	-0.53	-0.37	-0.86					**-0.14**

orf19.7518	0.79	1.37	0.27	1.93	1.25	-1.40	-0.36	-0.42	1.02		**0.79**
orf19.217	-1.20	-1.57	-3.40	-2.94	-1.35	-1.55	-2.02	-2.23			**-1.80**
orf19.255	-1.12	-1.07	-0.82	-2.07							**-1.10**
orf19.431	0.29	0.74	-0.77	0.50	-2.94						**0.29**
orf19.837.1	-0.30	0.16	-1.05	-0.57	-1.88						**-0.57**
orf19.1032	-2.06	-1.04	0.15	-0.42	-0.48	-2.89	-2.72	-1.61			**-1.32**
orf19.1035	3.84	4.35	4.17	6.47	5.14	2.27	2.40	2.24	0.57		**3.84**
orf19.1274	0.96	-0.04	-0.04	0.90	-2.00	-1.83	-1.98				**-0.04**
orf19.1496	0.08	-0.36	0.61	2.63	-0.01	0.24	0.28				**0.24**
orf19.2745	-0.65	-1.11	-0.48	-0.22	0.50	0.55	0.44				**-0.22**
orf19.2753	-0.43	-0.24	-0.57	3.50							**-0.34**
orf19.2808	3.44	1.68	3.20	4.53							**3.32**
orf19.3625	-0.62	0.49	-1.01	0.57	1.97	0.18	0.62	2.23	2.54	1.88	**0.60**
orf19.4647	-2.14	0.10	0.59	1.93	-0.40						**0.10**
orf19.5249	-1.48	-5.84	0.35	2.55	1.31	-0.89	-0.44	0.06	0.05	-1.13	**-0.20**
orf19.5729	-0.33	-1.09	-0.70	0.35	-0.09						**-0.33**
orf19.5910	0.67	0.49	-0.15	-0.59	0.78	0.32	-0.56				**0.32**
orf19.6680	-1.04	0.32	1.97	2.26							**1.14**
orf19.6781	0.82	0.11	-0.01	-0.17							**0.05**
orf19.6817	1.80	1.65	1.69	1.79							**1.74**
orf19.6888	-0.77	-1.08	-1.31	-1.13	0.21	0.07	1.31				**-0.77**
orf19.3193	-0.64	-2.18	-0.63	-3.05	-0.52	-0.02	1.07				**-0.63**
orf19.7371	-1.01	-1.16	-0.57	-1.15	-1.03	1.15	0.75	1.59			**-0.79**
orf19.3187	-0.27	-0.64	-0.30	-2.71							**-0.47**
orf19.4573	0.46	-0.51	-0.49	-0.67							**-0.50**
orf19.1718	-0.34	-0.80	-1.48	-1.06	-1.33						**-1.06**
orf19.2315	-2.21	-2.98	-2.77	-2.83	-2.80						**-2.80**
orf19.2612	-0.59	0.38	1.07	-0.22	-0.08						**-0.08**
orf19.4767	-1.40	0.07	0.45	1.44	0.00						**0.07**
orf19.4649	1.07	3.16	2.93	-0.27	1.62	0.33	2.79	1.29	1.88	1.49	**1.56**
orf19.1150	0.15	-0.17	-1.32	-1.35							**-0.75**
orf19.1757	5.08	5.98	8.29	11.08							**7.14**
orf19.2743	-0.40	0.47	0.17	1.22	0.29	-0.95	-0.57	-1.57	0.19		**0.17**
orf19.3865	-3.94	-2.33	-1.72	-0.64	-0.86						**-1.72**
orf19.1255	unable to detect by qPCR										
orf19.3736	unable to detect by qPCR										
orf19.1227	unable to detect by qPCR										
orf19.5251	unable to detect by qPCR										
orf19.7583	unable to detect by qPCR										
