# Supplementary material for: Candida albicans Commensalism and Pathogenicity Are Intertwined Traits Directed by a Tightly Knit Transcriptional Regulatory Circuit
Source: PLoS Biol. 2013 Mar 19;11(3):e1001510. doi: 10.1371/journal.pbio.1001510 (PMC3601966; doi:10.1371/journal.pbio.1001510)
Supplement: Table S2 — Target genes selected for testing role in gut colonization and systemic infection. (PDF) [file pbio.1001510.s008.pdf]

Table S2. Target genes selected for testing role in gut colonization and systemic infection.

ORF	NAME	ANNOTATION
ORF19.1069	*RPN4*	Regulator of proteasome genes
ORF19.1354	*UCF1*	Uncharacterized
ORF19.1363		Uncharacterized
ORF19.2765	*PGA62*	Adhesin-like cell wall protein; putative GPI-anchor
ORF19.35		Uncharacterized
ORF19.3669	*SHA3*	Putative serine/threonine kinase involved in the adaptation to low concentrations of glucose
ORF19.3670	*GAL1*	Galactokinase
ORF19.3672	*GAL10*	UDP-glucose 4-epimerase
ORF19.4450.1		Uncharacterized
ORF19.4961	*STP2*	Activates transcription of genes encoding amino acid permeases
ORF19.5079	*CDR4*	Putative transporter of ATP-binding cassette (ABC) superfamily
ORF19.5636	*RBT5*	GPI-anchored cell wall protein involved in hemoglobin utilization
ORF19.5960	*NCE102*	Membrane protein involved in secretion of proteins that lack classical secretory signal sequences
ORF19.7053	*GAC1*	Putative regulatory subunit of serine/threonine phosphoprotein phosphatase 1 (which regulates glycogen synthase)
ORF19.7084	*DFI1*	Cell-surface associated glycoprotein; promotes activation of Cek1p in a matrix-dependent manner
ORF19.7085		Uncharacterized
ORF19.740	*HAP41*	Uncharacterized
ORF19.822		Similar to heat-shock proteins
