# Supplementary material for: Candida albicans Commensalism and Pathogenicity Are Intertwined Traits Directed by a Tightly Knit Transcriptional Regulatory Circuit
Source: PLoS Biol. 2013 Mar 19;11(3):e1001510. doi: 10.1371/journal.pbio.1001510 (PMC3601966; doi:10.1371/journal.pbio.1001510)
Supplement: Table S3 — Strains used in this study. (PDF) [file pbio.1001510.s009.pdf]

**Table S3. *C. albicans* strains used in this study.**

| Strain | Genotype | Source |
|---|---|---|
| SN152 | *ura3Δ::λimm434::URA3-IRO1 arg4::hisG his1::hisG leu2::hisG*<br>*ura3Δ::λimm434 arg4::hisG his1::hisG leu2::hisG* | [34] |
| SN250 | *ura3Δ::λimm434::URA3-IRO1 arg4::hisG his1::hisG leu2::hisG::CdHIS1*<br>*ura3Δ::λimm434 arg4::hisG his1::hisG leu2::hisG::CmLEU2* | [5] |
| JCP087 | *ura3Δ::λimm434::URA3-IRO1 arg4::hisG his1::hisG leu2::hisG HMS1-13XMYC-FRT*<br>*ura3Δ::λimm434 arg4::hisG his1::hisG leu2::hisG hms1Δ::CmLEU2* | This work |
| JCP117 | *ura3Δ::λimm434::URA3-IRO1 arg4::hisG his1::hisG leu2::hisG RTG1-13XMYC-FRT*<br>*ura3Δ::λimm434 arg4::hisG his1::hisG leu2::hisG rtg1Δ::CdHIS1* | This work |
| JCP122 | *ura3Δ::λimm434::URA3-IRO1 arg4::hisG his1::hisG leu2::hisG RTG3-13XMYC-FRT*<br>*ura3Δ::λimm434 arg4::hisG his1::hisG leu2::hisG rtg3Δ::CmLEU2* | This work |
| JCP168 | *ura3Δ::λimm434::URA3-IRO1 arg4::hisG his1::hisG leu2::hisG LYS144::AgTEF1p-NAT1-AgTEF1UTR-TDH3p-GFP-LYS144*<br>*ura3Δ::λimm434 arg4::hisG his1::hisG leu2::hisG lys144Δ::CdHIS1* | This work |
| JCP170 | *ura3Δ::λimm434::URA3-IRO1 arg4::hisG his1::hisG leu2::hisG LYS14::AgTEF1p-NAT1-AgTEF1UTR-TDH3p-GFP-LYS14*<br>*ura3Δ::λimm434 arg4::hisG his1::hisG leu2::hisG lys14Δ::CdHIS1* | This work |
| JCP178 | *ura3Δ::λimm434::URA3-IRO1 arg4::hisG his1::hisG leu2::hisG ZCF21::AgTEF1p-NAT1-AgTEF1UTR-TDH3p-GFP-ZCF21*<br>*ura3Δ::λimm434 arg4::hisG his1::hisG leu2::hisG zcf21Δ::CdHIS1* | This work |
| JCP270 | *ura3Δ::λimm434::URA3-IRO1 arg4::hisG his1::hisG leu2::hisG::CdHIS1 HMS1::AgTEF1p-NAT1-AgTEF1UTR-TDH3p-HMS1*<br>*ura3Δ::λimm434 arg4::hisG his1::hisG leu2::hisG::CmLEU2 HMS1* | This work |
| JCP271 | *ura3Δ::λimm434::URA3-IRO1 arg4::hisG his1::hisG leu2::hisG::CdHIS1 RTG1::AgTEF1p-NAT1-AgTEF1UTR-TDH3p-RTG1*<br>*ura3Δ::λimm434 arg4::hisG his1::hisG leu2::hisG::CmLEU2 RTG1* | This work |
| JCP272 | *ura3Δ::λimm434::URA3-IRO1 arg4::hisG his1::hisG leu2::hisG::CdHIS1 TYE7::AgTEF1p-NAT1-AgTEF1UTR-TDH3p-TYE7*<br>*ura3Δ::λimm434 arg4::hisG his1::hisG leu2::hisG::CmLEU2 TYE7* | This work |
| JCP152 | *ura3Δ::λimm434::URA3-IRO1 arg4::hisG his1::hisG leu2::hisG zcf21::YFP-ACTp-SAT1*<br>*ura3Δ::λimm434 arg4::hisG his1::hisG leu2::hisG ZCF21* | This work |
| JCP154 | *ura3Δ::λimm434::URA3-IRO1 arg4::hisG his1::hisG leu2::hisG lys144::YFP-ACTp-SAT1*<br>*ura3Δ::λimm434 arg4::hisG his1::hisG leu2::hisG LYS144* | This work |
| JCP156 | *ura3Δ::λimm434::URA3-IRO1 arg4::hisG his1::hisG leu2::hisG lys14::YFP-ACTp-SAT1*<br>*ura3Δ::λimm434 arg4::hisG his1::hisG leu2::hisG LYS14* | This work |
| JCP171 | *ura3Δ::λimm434::URA3-IRO1 arg4::hisG his1::hisG leu2::hisG RTG1::AgTEF1p-NAT1-AgTEF1UTR-TDH3p-YFP-RTG1*<br>*ura3Δ::λimm434 arg4::hisG his1::hisG leu2::hisG rtg1Δ::CdHIS1* | This work |
| JCP086 | *ura3Δ::λimm434::URA3-IRO1 arg4::hisG his1::hisG leu2::hisG hms1Δ::CdHIS1 rps10Δ::HMS1-SAT1*<br>*ura3Δ::λimm434 arg4::hisG his1::hisG leu2::hisG hms1Δ::CmLEU2 RPS10* | This work |
| JCP191 | *ura3Δ::λimm434::URA3-IRO1 arg4::hisG his1::hisG leu2::hisG rtg3Δ::CdHIS1 rps10Δ::RTG3-SAT1*<br>*ura3Δ::λimm434 arg4::hisG his1::hisG leu2::hisG rtg3Δ::CmLEU2 RPS10* | This work |
| JCP192 | *ura3Δ::λimm434::URA3-IRO1 arg4::hisG his1::hisG leu2::hisG orf19.3625Δ::CdHIS1 rps10Δ::ORF19.3625-SAT1*<br>*ura3Δ::λimm434 arg4::hisG his1::hisG leu2::hisG orf19.3625Δ::CmLEU2 RPS10* | This work |

| | | |
|---|---|---|
| JCP194 | *ura3Δ::λimm434::URA3-IRO1 arg4::hisG his1::hisG leu2::hisG rtg1Δ::CdHIS1 rps10Δ::RTG1-SAT1*<br>*ura3Δ::λimm434 arg4::hisG his1::hisG leu2::hisG rtg1Δ::CmLEU2 RPS10* | This work |
| JCP195 | *ura3Δ::λimm434::URA3-IRO1 arg4::hisG his1::hisG leu2::hisG lys144Δ::CdHIS1 rps10Δ::LYS144-SAT1*<br>*ura3Δ::λimm434 arg4::hisG his1::hisG leu2::hisG lys144Δ::CmLEU2 RPS10* | This work |
| JCP196 | *ura3Δ::λimm434::URA3-IRO1 arg4::hisG his1::hisG leu2::hisG tye7Δ::CdHIS1 rps10Δ::TYE7-SAT1*<br>*ura3Δ::λimm434 arg4::hisG his1::hisG leu2::hisG tye7Δ::CmLEU2 RPS10* | This work |
| JCP146 | *ura3Δ::λimm434::URA3-IRO1 arg4::hisG his1::hisG leu2::hisG orf19.1354Δ::CdHIS1*<br>*ura3Δ::λimm434 arg4::hisG his1::hisG leu2::hisG orf19.1354Δ::CmLEU2* | This work |
| JCP147 | *ura3Δ::λimm434::URA3-IRO1 arg4::hisG his1::hisG leu2::hisG orf19.2765Δ::CdHIS1*<br>*ura3Δ::λimm434 arg4::hisG his1::hisG leu2::hisG orf19.2765Δ::CmLEU2* | This work |
| JCP148 | *ura3Δ::λimm434::URA3-IRO1 arg4::hisG his1::hisG leu2::hisG orf19.3672Δ::CdHIS1*<br>*ura3Δ::λimm434 arg4::hisG his1::hisG leu2::hisG orf19.3672Δ::CmLEU2* | This work |
| JCP149 | *ura3Δ::λimm434::URA3-IRO1 arg4::hisG his1::hisG leu2::hisG orf19.4450.1Δ::CdHIS1*<br>*ura3Δ::λimm434 arg4::hisG his1::hisG leu2::hisG orf19.4450.1Δ::CmLEU2* | This work |
| JCP150 | *ura3Δ::λimm434::URA3-IRO1 arg4::hisG his1::hisG leu2::hisG orf19.5636Δ::CdHIS1*<br>*ura3Δ::λimm434 arg4::hisG his1::hisG leu2::hisG orf19.5636Δ::CmLEU2* | This work |
| JCP151 | *ura3Δ::λimm434::URA3-IRO1 arg4::hisG his1::hisG leu2::hisG orf19.7085Δ::CdHIS1*<br>*ura3Δ::λimm434 arg4::hisG his1::hisG leu2::hisG orf19.7085Δ::CmLEU2* | This work |
| JCP160 | *ura3Δ::λimm434::URA3-IRO1 arg4::hisG his1::hisG leu2::hisG orf19.3669Δ::CdHIS1*<br>*ura3Δ::λimm434 arg4::hisG his1::hisG leu2::hisG orf19.3669Δ::CmLEU2* | This work |
| JCP161 | *ura3Δ::λimm434::URA3-IRO1 arg4::hisG his1::hisG leu2::hisG orf19.3670Δ::CdHIS1*<br>*ura3Δ::λimm434 arg4::hisG his1::hisG leu2::hisG orf19.3670Δ::CmLEU2* | This work |
| JCP162 | *ura3Δ::λimm434::URA3-IRO1 arg4::hisG his1::hisG leu2::hisG orf19.7084Δ::CdHIS1*<br>*ura3Δ::λimm434 arg4::hisG his1::hisG leu2::hisG orf19.7084Δ::CmLEU2* | This work |
| JCP163 | *ura3Δ::λimm434::URA3-IRO1 arg4::hisG his1::hisG leu2::hisG orf19.740Δ::CdHIS1*<br>*ura3Δ::λimm434 arg4::hisG his1::hisG leu2::hisG orf19.740Δ::CmLEU2* | This work |
| JCP164 | *ura3Δ::λimm434::URA3-IRO1 arg4::hisG his1::hisG leu2::hisG orf19.35Δ::CdHIS1*<br>*ura3Δ::λimm434 arg4::hisG his1::hisG leu2::hisG orf19.35Δ::CmLEU2* | This work |
| JCP165 | *ura3Δ::λimm434::URA3-IRO1 arg4::hisG his1::hisG leu2::hisG orf19.5079Δ::CdHIS1*<br>*ura3Δ::λimm434 arg4::hisG his1::hisG leu2::hisG orf19.5079Δ::CmLEU2* | This work |
| JCP166 | *ura3Δ::λimm434::URA3-IRO1 arg4::hisG his1::hisG leu2::hisG orf19.5960Δ::CdHIS1*<br>*ura3Δ::λimm434 arg4::hisG his1::hisG leu2::hisG orf19.5960Δ::CmLEU2* | This work |
| JCP181 | *ura3Δ::λimm434::URA3-IRO1 arg4::hisG his1::hisG leu2::hisG orf19.822Δ::CdHIS1*<br>*ura3Δ::λimm434 arg4::hisG his1::hisG leu2::hisG orf19.822Δ::CmLEU2* | This work |
| JCP190 | *ura3Δ::λimm434::URA3-IRO1 arg4::hisG his1::hisG leu2::hisG orf19.7053Δ::CdHIS1*<br>*ura3Δ::λimm434 arg4::hisG his1::hisG leu2::hisG orf19.7053Δ::CmLEU2* | This work |
