# Supplementary material for: Candida albicans Commensalism and Pathogenicity Are Intertwined Traits Directed by a Tightly Knit Transcriptional Regulatory Circuit
Source: PLoS Biol. 2013 Mar 19;11(3):e1001510. doi: 10.1371/journal.pbio.1001510 (PMC3601966; doi:10.1371/journal.pbio.1001510)
Supplement: Table S4 — Primers used in this study. (PDF) [file pbio.1001510.s010.pdf]

Table S4. Primers used in this study

Name	Description	Sequence (5' to 3')
JCP416	del orf19.1354 pr1	CCCCCTTGAATTGATTGTTG
JCP417	del orf19.1354 pr3	CACGGCGCGCCTAGCAGCGGCCGCCATGATCGTTAATTTG
JCP418	del orf19.1354 pr4	GTCAGCGGCCGCATCCCTGCTTGTTACTGCTGCTGCTGGT
JCP419	del orf19.1354 pr6	CTGAATTGGACGGAGACGTA
JCP420	del orf19.1354 test 3'	CCATCGTCGATAAACCTTAATTTC
JCP421	del orf19.1363 pr1	AGTGGTGTGGGCTTTGAAAC
JCP422	del orf19.1363 pr3	CACGGCGCGCCTAGCAGCGGTTTAAAGAAAACAGAAAAGAAGAATTG
JCP423	del orf19.1363 pr4	GTCAGCGGCCGCATCCCTGCACATGGCAAGGTTGGCTAAT
JCP424	del orf19.1363 pr6	AAATTTCCCTTCCAAAACAAA
JCP425	del orf19.1363 test 3'	ACAACCTCTGGCCCCATT
JCP426	del orf19.2765 pr1	CATGCATGTAACCCACTTCG
JCP427	del orf19.2765 pr3	CACGGCGCGCCTAGCAGCGGAGCGGATGAGAATTGCATTG
JCP428	del orf19.2765 pr4	GTCAGCGGCCGCATCCCTGCCCCTTTCCCTTTCTTCATTCT
JCP429	del orf19.2765 pr6	CAGAATCATTGAATACACCGAAAT
JCP430	del orf19.2765 test 3'	TGAAAGCTCGGATGAAGTGA
JCP431	del orf19.35 pr1	TTCTGTTCCCTTTCCCTTTTC
JCP432	del orf19.35 pr3	CACGGCGCGCCTAGCAGCGGTCCATCTGAGGAGGATAACTTGA
JCP433	del orf19.35 pr4	GTCAGCGGCCGCATCCCTGCACCGGGTTATACATGTGAATG
JCP434	del orf19.35 pr6	TGGTTCAAAACTTTGTTGGATG
JCP435	del orf19.35 test 3'	TTCTTTAGAAATTGGCCCCTTA
JCP436	del orf19.3669 pr1	GGAATGAAAATGAGAATGAGAAAA
JCP437	del orf19.3669 pr3	CACGGCGCGCCTAGCAGCGGACAAAAGGGAAGGGGGTAAA
JCP438	del orf19.3669 pr4	GTCAGCGGCCGCATCCCTGCTCAAAGTGTTGAATACTGTTAAGTTGT
JCP439	del orf19.3669 pr6	CTGTTTGGGTTGCTTTTCGT
JCP440	del orf19.3669 test 3'	CACCAACAAACAACCCAACA
JCP441	del orf19.3670 pr1	TTTGTCGGGCAAAGAAAAAC
JCP442	del orf19.3670 pr3	CACGGCGCGCCTAGCAGCGGTCAAACGTAGGAACTGACATGG
JCP443	del orf19.3670 pr4	GTCAGCGGCCGCATCCCTGCGGCGACGAATATCGCAACTA
JCP444	del orf19.3670 pr6	TGACTTGTATAAGCCTACTTTGCTTT
JCP445	del orf19.3670 test 3'	TTGGGTCCGGAATAGTATGG
JCP446	del orf19.3672 pr1	ATCATCCATCCGAACGTTTT
JCP447	del orf19.3672 pr3	CACGGCGCGCCTAGCAGCGGTTTTGGAAGGACTTGAATTTATCA
JCP448	del orf19.3672 pr4	GTCAGCGGCCGCATCCCTGCAGCCATTGATAGCTTGTTGTTG
JCP449	del orf19.3672 pr6	CAATTTAGAACCAATGGCATGA
JCP450	del orf19.3672 test 3'	TATTTGCACTCCCGGCTTTA
JCP451	del orf19.4450.1 pr1	GTCGTGGAGAGCAGGAAGTG
JCP452	del orf19.4450.1 pr3	CACGGCGCGCCTAGCAGCGGCCACTTCCGAAATTGAAAAGG
JCP453	del orf19.4450.1 pr4	GTCAGCGGCCGCATCCCTGCAAGTCACCCAAGCCATGTTT

JCP454	del orf19.4450.1 pr6	GCTATGCCCTTGACTTGTCC
JCP455	del orf19.4450.1 test 3'	TGGCAATGAAAACAAAAACG
JCP456	del orf19.5079 pr1	AGGCTAGCAGTTTGAGCAAGA
JCP457	del orf19.5079 pr3	CACGGCGCGCCTAGCAGCGGCGGCATCTGCCATAGCTAAA
JCP458	del orf19.5079 pr4	GTCAGCGGCCGCATCCCTGCTGCAGATAAATGCCAAGTGC
JCP459	del orf19.5079 pr6	AGGATCATTCGACACCAAGC
JCP460	del orf19.5079 test 3'	AAAGCATGAGTCCCGACAAC
JCP461	del orf19.5636 pr1	CCTCGAGTAATTAAGCCAAGC
JCP462	del orf19.5636 pr3	CACGGCGCGCCTAGCAGCGGACGGAAGTCATGGAAAATGC
JCP463	del orf19.5636 pr4	GTCAGCGGCCGCATCCCTGCGCTCCCTTCCTTGCTATTGTC
JCP464	del orf19.5636 pr6	TTGGTATGGGTTATGATTCCTG
JCP465	del orf19.5636 test 3'	GGATTGGCACTGCAAGTGTA
JCP466	del orf19.5960 pr1	TCCCCTTCATAACAAGAGAAAAA
JCP467	del orf19.5960 pr3	CACGGCGCGCCTAGCAGCGGTGAATTCAAAAACAAGAAAGGAGA
JCP468	del orf19.5960 pr4	GTCAGCGGCCGCATCCCTGCCCCAAGTTTAAGATTTGGTTTGAA
JCP469	del orf19.5960 pr6	CCCAAAATAAACACAGCTTGA
JCP470	del orf19.5960 test 3'	CACCTCAGCACAGCAAGGTA
JCP471	del orf19.7053 pr1	TTAACAACCACCACCCATCA
JCP472	del orf19.7053 pr3	CACGGCGCGCCTAGCAGCGGCGATTGATTGATTGATTGATTGA
JCP473	del orf19.7053 pr4	GTCAGCGGCCGCATCCCTGCTCTTTCCATTCCATTCATTCTTT
JCP474	del orf19.7053 pr6	TTGTTTTCAATTGTTGCCAGA
JCP475	del orf19.7053 test 3'	GTGGAGCTTGGGTAGACGAG
JCP476	del orf19.7084 pr1	ATGGTGGTGGCGGTAATAAA
JCP477	del orf19.7084 pr3	CACGGCGCGCCTAGCAGCGGAAAACGAACGTGGCAGAAGT
JCP478	del orf19.7084 pr4	GTCAGCGGCCGCATCCCTGCTTTGGATGAGGGTGGTTTTT
JCP479	del orf19.7084 pr6	TTGGAATGGTTCATTTGTCG
JCP480	del orf19.7084 test 3'	GTGCTGTTTGTCCGTTGAAA
JCP481	del orf19.7085 pr1	AAAGGGGTGATACTCGTTGC
JCP482	del orf19.7085 pr3	CACGGCGCGCCTAGCAGCGGAGGGAAAAATGAAGGGAATG
JCP483	del orf19.7085 pr4	GTCAGCGGCCGCATCCCTGCTGGTAGCTGTCGCGTCTAAA
JCP484	del orf19.7085 pr6	ACGATTCAAACCCCATTGAC
JCP485	del orf19.7085 test 3'	TCGGGATAATCGACAGAAGC
JCP486	del orf19.740 pr1	TGATGTGTCTATTAACGCATATGTTG
JCP487	del orf19.740 pr3	CACGGCGCGCCTAGCAGCGGTTGAGTGAACTAGTGGGAAAAGAA
JCP488	del orf19.740 pr4	GTCAGCGGCCGCATCCCTGCTTTGATGTGATGTGATGTGATGA
JCP489	del orf19.740 pr6	AGAGATGAGCTGCAAAATGGA
JCP490	del orf19.740 test 3'	TTCCGTGAGACACACCAGAG
JCP491	del orf19.822 pr1	TGAGTGATTGCTTTGCATCG
JCP492	del orf19.822 pr3	CACGGCGCGCCTAGCAGCGGCAAAACCATAGGAAGGGTGAA
JCP493	del orf19.822 pr4	GTCAGCGGCCGCATCCCTGCCCCCCCTTTATTTGTTTTTGGT
JCP494	del orf19.822 pr6	CCGGTTATGCTCGAAAACAT
JCP495	del orf19.822 test 3'	TGGATTTTCGTTGTGAGTGG

JCP496	signature_tag3 pr5	GCAGGGATGCGGCCGCTGACATAGGGCTGTCATGATAAGGGCTCGGATCCACTAGTAACG
JCP497	signature_tag5 pr5	GCAGGGATGCGGCCGCTGACAGCGATAACACCTTCATGAGGCTCGGATCCACTAGTAACG
JCP498	signature_tag7 pr5	GCAGGGATGCGGCCGCTGACTAATAACGTCGCATGCCATGGCTCGGATCCACTAGTAACG
JCP499	signature_tag8 pr5	GCAGGGATGCGGCCGCTGACTTTTGTGGAGCCTTTCATGAGCTCGGATCCACTAGTAACG
JCP500	signature_tag9 pr5	GCAGGGATGCGGCCGCTGACAAATCTGTTTGGCATGACGAGCTCGGATCCACTAGTAACG
JCP501	signature_tag10 pr5	GCAGGGATGCGGCCGCTGACCCACCCGCATCAAGAAATGTGCTCGGATCCACTAGTAACG
JCP502	signature_tag11 pr5	GCAGGGATGCGGCCGCTGACAAGACAATGACGATGAAGGTGCTCGGATCCACTAGTAACG
JCP503	signature_tag12 pr5	GCAGGGATGCGGCCGCTGACACACCGCAACCTGATTGATAGCTCGGATCCACTAGTAACG
JCP504	signature_tag18 pr5	GCAGGGATGCGGCCGCTGACCCACATGCTCCTGCAACTGCGCTCGGATCCACTAGTAACG
JCP505	signature_tag20 pr5	GCAGGGATGCGGCCGCTGACGTCTTGCTCGAGAGTCCAGAGCTCGGATCCACTAGTAACG
JCP506	signature_tag23 pr5	GCAGGGATGCGGCCGCTGACTCTAGACCCTCCTTCTTGGCGCTCGGATCCACTAGTAACG
JCP507	signature_tag34 pr5	GCAGGGATGCGGCCGCTGACATCGACTGTTCTATTGGCAAGCTCGGATCCACTAGTAACG
JCP508	signature_tag36 pr5	GCAGGGATGCGGCCGCTGACCCTGACTGTCTAATCGGCAAGCTCGGATCCACTAGTAACG
JCP509	signature_tag37 pr5	GCAGGGATGCGGCCGCTGACTGATAAGCATGGCTCATTGCGCTCGGATCCACTAGTAACG
JCP510	signature_tag44 pr5	GCAGGGATGCGGCCGCTGACATAAGTCCGTGCCGAAAACTGCTCGGATCCACTAGTAACG
JCP511	signature_tag45 pr5	GCAGGGATGCGGCCGCTGACAAAGGTATGGTTTAGCTGATGCTCGGATCCACTAGTAACG
JCP527	del orf19.1354 test int F	TGAATCGTTTGTTCGTGATGA
JCP528	del orf19.1354 test int R	CATCCAATTCCAATCTCCAA
JCP529	del orf19.1363 test int F	ATTCGCACGTCTCATCTCCT
JCP530	del orf19.1363 test int R	GCAACATTAGCAACGGTGTG
JCP531	del orf19.2765 test int F	TGGCTGCTTACTCCAACTCC
JCP532	del orf19.2765 test int R	GGGGAAGATTCAGCTGGTTT
JCP533	del orf19.35 test int F	AACACCCACACCAACACCTT
JCP534	del orf19.35 test int R	TTGAAAAATGTCCCCAACCT
JCP535	del orf19.3669 test int F	TGGATCATTTTGAGCAAGGA
JCP536	del orf19.3669 test int R	ATGACGATCCACGACATGAA
JCP537	del orf19.3670 test int F	CCAAGAATTTTGCCAAGGAA
JCP538	del orf19.3670 test int R	CACATTGATCCATTCCACCA
JCP539	del orf19.3672 test int F	TGGTGCAAATGGATTCGATA
JCP540	del orf19.3672 test int R	TGGGCTAGCAGCTTTTTCAT
JCP541	del orf19.4450.1 test int F	TGGCTTTAGCAAAATCTAAATTACT
JCP542	del orf19.4450.1 test int R	GCATTGGGTGAACTCTTTCG
JCP543	del orf19.5079 test int F	ACGTTTGAACCCGAACTCAC
JCP544	del orf19.5079 test int R	TCAAAAATGTCGAGCAACCA
JCP545	del orf19.5636 test int F	ATCTGCTGGTGTTTGGGAAC
JCP546	del orf19.5636 test int R	GCTTCAACGGAAACAGAAGC
JCP547	del orf19.5960 test int F	GCTTGGCCAGTGATTTTGTT
JCP548	del orf19.5960 test int R	GAAAGGGAAATGGCACTGAA
JCP549	del orf19.7053 test int F	ACAGAAACCATTGCCAGACC
JCP550	del orf19.7053 test int R	CGAAGACGACGATTTGGTTT
JCP551	del orf19.7084 test int F	CAACAACATCGGCTTCTCAA
JCP552	del orf19.7084 test int R	TGACACCCAATTCACCATTG

JCP553	del orf19.7085 test int F	TTGCTTGTTAGGCTGCAATG
JCP554	del orf19.7085 test int R	TAGCCTTGCCATTCAAATCC
JCP555	del orf19.740 test int F	AAACGCACACCCACCTTTAC
JCP556	del orf19.740 test int R	TTAATTGATGCGCACATGGT
JCP557	del orf19.822 test int F	CTCACAGAGCATTGGCAAGA
JCP558	del orf19.822 test int R	GCAGCTGCTTTGGAAATAGG
JCP052	HMS1 MYC tag F	GAAACAAGAACATGATGAATTGGTATTAAAGGCAAGAATGTTAGGTTTAGTTATTGATGAAGATATGCGGATCCCCGGGTTAATTAACGG
JCP053	HMS1 MYC tag R	TTCATTAACGTAAATTTCAAACCAAAACTTATACATAAAAATTCTAAAAACTAGAACCACCCAAGGCGGCCGCTCTAGAACTAGTGGATC
JCP330	RTG1 MYC tag F	TTTCAAGAACGTGTTAATCAAAAGTGCATCCACAAGTAAATCAGGTAGACGGGGAAGTACGAGTGGACGGATCCCCGGGTTAATTAACGG
JCP331	RTG1 MYC tag R	CAAATGATTATAGAAAATAGAAAATAGAAAAAATAAACACTATAACAGTCAGTGAGTTTCTTTTGGCGGCCGCTCTAGAACTAGTGGATC
JCP358	RTG3 MYC tag F	CAGAGAAATTGCCAACTACAATCCAGAAGATTTTTTTGCAGATATAGGTACTAGTACTGAGAATATCCGGATCCCCGGGTTAATTAACGG
JCP359	RTG3 MYC tag R	AAAGTAAACGTCATAATAATAATACTTATACTTATACTACTACCTCCAGAAATTAAAATCGTAAGGCGGCCGCTCTAGAACTAGTGGATC
JCP408	TDH3p-LYS144-GFP F	GTACGTGAACACATAAAGATCTAGCCTAAGATTGACATACATATTATTATTAAAAATCCCCAATGACCGCATCAAGCTTGCCTCGTCCCC
JCP560	TDH3p R	GAATAATTCTTCACCTTTAGACATATTTGAATTCAATTGTGATG
JCP561	GFP F	ATGTCTAAAGGTGAAGAATTATTC
JCP567	GFP-LYS144 R	GGTGGTAGCTTCTTGTTGTGAAGTTTCTCCTAATGGTGATTTTGTGGGGTTTGGGCTAGATGAACCTTTGTACAATTCATCCATACCATG
JCP410	TDH3p-LYS14-GFP F	CAGAATTGTTTATTTGTTTTTGTTTTACTTGGCTAAAGCTTTGTTCTGCTTTGTTTTGTTTGTTGTTCCTATCAAGCTTGCCTCGTCCCC
JCP568	GFP-LYS14 R	ATCCAGTGGAGAATTAACACCCGAAGAAGACGCGGTAGCAGGTGATAAAGAAGATGGTGATTGTGATTTGTACAATTCATCCATACCATG
JCP404	TDH3p-ZCF21-GFP F	AGTGGGCAGAAAAATACGTACAAAAGTAAACACTTGTTATTATTTTATTATCTAATTCTCTCTTTCTTTCATCAAGCTTGCCTCGTCCCC
JCP578	GFP-ZCF21 R	AGTAGTTGTTGATGCAGGTTTTTTGGGAGGATCAGGGTTCTTGAGAGCCTTCTGATAAATATCCATTTTGTACAATTCATCCATACCATG
JCP512	replace ZCF21 5' YFP F	CATCTTTCATTCTTTTTAGTTTCCCCCAATTAACTTTCAAATATAATTTACTATACTGTTCCAAAGATGTCTAAAGGTGAAGAATTATTC
JCP513	YFP R	CAAAACCAGATTTCCAGATTTCCAGTTATTTGTACAATTCATCCATACC
JCP514	YFP F	CTGGAAATCTGGAAATCTGGTTTTG
JCP515	replace ZCF21 3' SAT1 R	AAAAAAAGGAAAATGCCATGCCAGCATATATATGCATATATAGAGAAATACTATAATACTATATCCATTAGGCGTCATCCTGTGCTCCCG
JCP516	replace LYS144 5' YFP F	TATGAAAGGCATCATTATTCCTTTCCATAACTCAAAACCAATTACAGTAATTGAAGATAAGATCAAATGTCTAAAGGTGAAGAATTATTC
JCP517	replace LYS144 3' SAT1 R	TAACAAAACAAAATCTATAAAACCGTGACACACAAAACTAAACTTACACTTACAAGCAATATCTATTTTAGGCGTCATCCTGTGCTCCCG

JCP518	replace LYS14 5' YFP F	CATCTCAATCAACCAACTATCATCCCAACCACTACAACTATTCATAAACCAAGTACTGAC TTCGTCATGTCTAAAGGTGAAGAATTATTC
JCP519	replace LYS14 3' SAT1 R	ATAAAGAACTAAAACAGTACGTACAGACTTTTAAATGTTTTCCTAATTGTAGACAACCA AATATTTTTTAGGCGTCATCCTGTGCTCCCG
JCP559	TDH3p-RTG1-GFP F	AACCGCTGGAAGCCTTAGAAAACAATAGATACCATTGACTGTGACACTTATTTTTTACA CAATTAGATAAATCAAGCTTGCCTCGTCCCC
JCP562	GFP-RTG1 R	CAAGTCGGTATCATATTGTTGGTTATTCTCAACCAGATATGTATCATAAAACTCTCCGA ATTGAGATTTGTACAATTCATCCATACCATG
JCP267	TDH3p-HMS1 F	CACTTGATAAGTCTTGCTTTTAAGTTCAGGCTCAAGAAAAGAAATCTCAAATATAAAA TTAGTATAAATAATCAAGCTTGCCTCGTCCCC
JCP771	TDH3p-HMS1 R	GTAATTGTTCATTATTTTTATCAAACAAGGAAGTTAAAAATGCGTTATAATCATTCAAT TTAAATATCATATTTGAATTCAATTGTGATG
JCP705	TDH3p-RTG1 F	GTCATATTTATTGTAGATACGCACACGACAATACATGTATATATTATCTAATAGTAACT ATAAAACATTTATCAAGCTTGCCTCGTCCCC
JCP772	TDH3p-RTG1 R	TCAAGTCGGTATCATATTGTTGGTTATTCTCAACCAGATATGTATCATAAAACTCTCCG AATTGAGACATATTTGAATTCAATTGTGATG
JCP774	TDH3p-TYE7 F	CATTGTAATTTGAAGATAAGTGATCGATTTATGTTTAAGGTGTTAATGAATTTGTTTGG GTCACAAAATCATCAAGCTTGCCTCGTCCCC
JCP775	TDH3p-TYE7 R	TATATTCATTCATGACATTGTTGTTGTTGTTGTTAGCGTTCAATTGATTTTCTTGCTG GAATGAACTCATATTTGAATTCAATTGTGATG
JCP794	qPCR_hms1_F	ACAAAGACACCAATCTCATCAA
JCP795	qPCR_hms1_R	TGGAGGAGGAGCTAAACCAA
JCP796	qPCR_rtg1_F	CCAAACCACAATCCAGAAGAA
JCP797	qPCR_rtg1_R	TCGTTGCCAACATCTTTATCA
JCP798	qPCR_tye7_F	TTGGATGAACGGTTTGATTG
JCP799	qPCR_tye7_R	TTTGCGCAATACTGTCTGATG
JCP802	qPCR_zcf21_F	TCCCACCCATGAACCATAAT
JCP803	qPCR_zcf21_R	TTGGGGCTATTAGTGCTGCT
JCP805	qPCR_gal10_F	TGGTGGTGCAGGTTACATTG
JCP806	qPCR_gal10_R	GCAGCAAAATGAATGACTCC
JCP807	qPCR_dfi1_F	CGACCAGACCAAGCCATTAT
JCP808	qPCR_dfi1_R	TTTCAAGGGACACTGTGGTG
JCP809	qPCR_hap41_F	GAAAATACCACCGCAACCAC
JCP810	qPCR_hap41_R	CGTCGATATTGGCTGACAAA
JCP811	qPCR_nce102_F	CCACCACCATTACCCAACAT
JCP812	qPCR_nce102_R	GCAAATGCTGCAACGAAGTA
JCP813	qPCR_act1_F	AAGCCCAATCCAAAAGAGGT
JCP814	qPCR_act1_R	GGAGCTTCGGTCAACAAAAC
